# Feasibility and acceptability of a milk and resistance exercise intervention to improve muscle function in community-dwelling older adults (MIlkMAN): Pilot study

**DOI:** 10.1371/journal.pone.0235952

**Published:** 2020-07-10

**Authors:** Antoneta Granic, Christopher Hurst, Lorelle Dismore, Emma Stevenson, Avan A. Sayer, Terry Aspray

**Affiliations:** 1 AGE Research Group, Translational and Clinical Research Institute, Faculty of Medical Sciences, Newcastle University, Newcastle upon Tyne, United Kingdom; 2 NIHR Newcastle Biomedical Research Centre, Newcastle upon Tyne Hospitals NHS Foundation Trust and Newcastle University, Newcastle upon Tyne, United Kingdom; 3 Northumbria Healthcare NHS Foundation Trust, Research and Development, North Tyneside General Hospital, North Shields, United Kingdom; 4 Population Health Sciences Institute, Faculty of Medical Sciences, Newcastle University, Newcastle upon Tyne, United Kingdom; 5 Human Nutrition Research Centre, Newcastle University, Newcastle upon Tyne, United Kingdom; 6 Musculoskeletal Unit, Newcastle upon Tyne Hospitals NHS Foundation Trust, Freeman Hospital, Newcastle upon Tyne, United Kingdom; IRCCS E. Medea, ITALY

## Abstract

**Background:**

Dietary protein supplementation combined with resistance exercise (RE) may counteract declines in muscle strength, mass, and function (sarcopenia), but the role of whole foods rich in protein, such as milk, is less well understood. In the MIlkMAN study, we aimed to examine the feasibility and acceptability of milk+RE as an intervention for muscle function in community-dwelling older adults, and provide exploratory pilot data for future substantive research in population at risk of sarcopenia.

**Methods:**

In a parallel groups design, 30 older adults (71.7±3.6 years; 12 women) were randomised into three groups: WM (whole milk 3.6% fat)+RE, SM (skimmed milk 0.3% fat)+RE, and C (isocaloric carbohydrate drink)+RE. RE was performed twice-weekly over 6 weeks in a community gym, followed by the consumption of 500 ml of milk (~20 g protein) or carbohydrate drink immediately after exercise and a further 500 ml at home within the following 4–5 hours. The feasibility and acceptability of the study was determined by calculating recruitment and attendance rates, compliance with the intervention, rating participants’ experiences, and recording adverse health events.

**Results:**

The response rate was 49% (out of 400 invitations sent), and the recruitment rate was 73.2% (30 participants recruited out of 41 screened for eligibility). Twenty-nine participants completed the intervention—an attendance rate of 97.1%; 89.7% rated their experience as ‘excellent’/very good’. Compliance with taking the drinks was 97.1% (WM), 98.3% (SM), and 95.0% (C); 93.1% rated their drink intake as ‘easy’/’very easy’ with no adverse effects. Collection of exploratory pilot data to inform future trials was successful. Mean change in grip strength, 5-chair rises, and gait speed were 0.9±3.4 kg, 1.8±2.2 s, 0.1±0.1 m/s, respectively with no differences between the groups.

**Conclusions:**

This community-based milk+RE intervention was feasible and acceptable to older adults. The study successfully collected pilot data for future substantive research.

## Introduction

Sarcopenia is a muscle disorder commonly but not exclusively seen in older adults, characterised by a decline in skeletal muscle strength and mass, leading to impaired function [1,2] and adverse health outcomes, including falls, frailty, poor quality of life (QoL), hospitalisation, and mortality [3]. Direct and indirect health care costs of sarcopenia are substantially increased [4], emphasising the need for interventions to optimise and preserve muscle health and function in populations at risk [5,6]. Specifically, intervention studies combining protein supplementation with resistance exercise (RE) have been shown to ameliorate the decline in muscle mass and function in older adults at risk of sarcopenia and frailty [7,8]. However, these studies have predominantly involved protein supplements (e.g. whey) or isolated nutrients (e.g. leucine) and less is known about the role of whole foods rich in protein, such as milk and milk products, in sarcopenia [9,10]. Incorporating foods dense in nutrients beneficial for ageing muscle [11] within a balanced diet may inform the development of strategies to mitigate sarcopenia that are acceptable to older adults [12], and not reliant on nutritional supplements or pharmacotherapy.

Cow’s milk can be an important part of a healthy diet and a source of several essential nutrients, including high-quality proteins such as whey (a rich source of branched-chain amino acid leucine (5.6 g per 25 g of whey protein [13]), the main dietary regulator of muscle protein synthesis (MPS)), bioactive lipids and fatty acids, minerals, and vitamins [14], which synergistically may be beneficial for skeletal muscle (discussed in Granic et al. 2020 [15]). In addition, the benefit of milk proteins is thought to come from the higher essential amino acid (EAA) score, bioavailability and solubility compared with other animal and plant protein sources [16]. Specifically, milk (whey) total protein has 49% (52%) of EAA and 10.9% (13.4%) of leucine compared with 44% EAA (8.8% leucine) in beef, and 38% EAA (8.0% leucine) in soy [16]. Milk bioactive peptides produced by enzymatic hydrolysis have multiple biological functions, including antioxidant, anti-inflammatory, antihypertensive and immunomodulatory effects, as well as aiding the absorption of other nutrients [14,17]. For example, in one study of young adults consuming milk after exercise, whole milk (with a high fat content) showed an enhanced utilisation of EAA for MPS when compared with those consuming fat-free milk [18]. Exercise-induced damage, muscle soreness, and decline in post-exercise performance were reduced in men and women consuming whole milk instead of an isocaloric carbohydrate drink after exercise [19]. Thus, the nutritional value of whole milk for MPS may extend beyond EAA to milk fats (a delivery medium for fat-soluble vitamins A, D and E) [14], minerals and carbohydrates, and the ratio of EAA to fat in milk may be relevant for ageing muscle.

Because of a blunted response to protein ingestion post-exercise, especially to lower amounts (<20 g protein, <10 g of EAA), older adults may require a higher protein intake and repeated protein feeding for MPS compared to younger adults [20,21]. Milk may serve as a nutritious, easy to prepare, and affordable dietary source to support muscle health in later life, although little is known about the impact of milk providing >20 g of protein or the effect of different milk fat contents after exercise on muscle in older adults at risk of sarcopenia.

The aim of the present investigation was to evaluate the feasibility and acceptability of consumption of whole (3.6% fat) or skimmed milk (0.3% fat) or isocaloric carbohydrate drink after RE as an intervention in community-dwelling older adults aged ≥65 years. The following questions were addressed: Is an intervention 2×500 ml milk+RE twice-weekly for 6 weeks (1) feasible and (2) acceptable to older adults, and (3) able to provide exploratory pilot data for future substantive research.

## Methods

The protocol for the MIlk Intervention Muscle AgeiNg (MIlkMAN) study has been described in detail previously [22]. The study was prospectively registered at https://www.isrctn.com/ISRCTN13398279 and was reported in accordance with the Consolidated Standards of Reporting Trials (CONSORT) 2010 statement extension for reporting randomised pilot and feasibility trials [23].

### Ethics

The study was approved by North East–Newcastle and North Tyneside Research Ethics Committee 1 (Reference: 18/NE/0265) and conformed to the principles of the Declaration of Helsinki. Written informed consent was obtained from all participants prior to enrolment.

### Study design

MIlkMAN was a three-arm, parallel groups pilot study. All participants performed a 6-week community-based RE programme consisting of two exercise sessions per week. Participants were allocated to consume a bolus intake of either (1) whole milk (3.6% fat; WM+RE group), (2) skimmed milk (0.3% fat; SM+RE group) or (3) control drink (isocaloric carbohydrate drink; C+RE group) following each RE session. We used the three groups for the following reasons: (1) to assess the feasibility and acceptability of WM and SM intake owing their differences in caloric value, fat content, taste, palatability, and older adults’ milk preferences; (2) to gather information about the feasibility and acceptability of WM intake to aid the development of future research testing the hypothesis whether milk fats enhance the utilisation of EAA for MPS [18], and faster functional muscle recovery in older adults as observed in young adults [19], and (3) to assess the feasibility and acceptability of isocaloric carbohydrate drink (matched to the caloric value of WM) for comparison across the groups, and to exclude the effect of energy on muscle-related outcomes in future substantive research.

### Participants

#### Sample size

Determination of the sample size was guided by the need to fulfil the primary aim of the study (i.e. the feasibility and acceptability of the intervention), practical feasibility, and funding limitations. We aimed to recruit 30 participants (10 participants per group; 5 men and 5 women in each group) as it has been previously suggested that this number may be sufficient to inform feasibility and to plan for a larger study [24,25].

#### Eligibility, recruitment and setting

**Fig 1:**
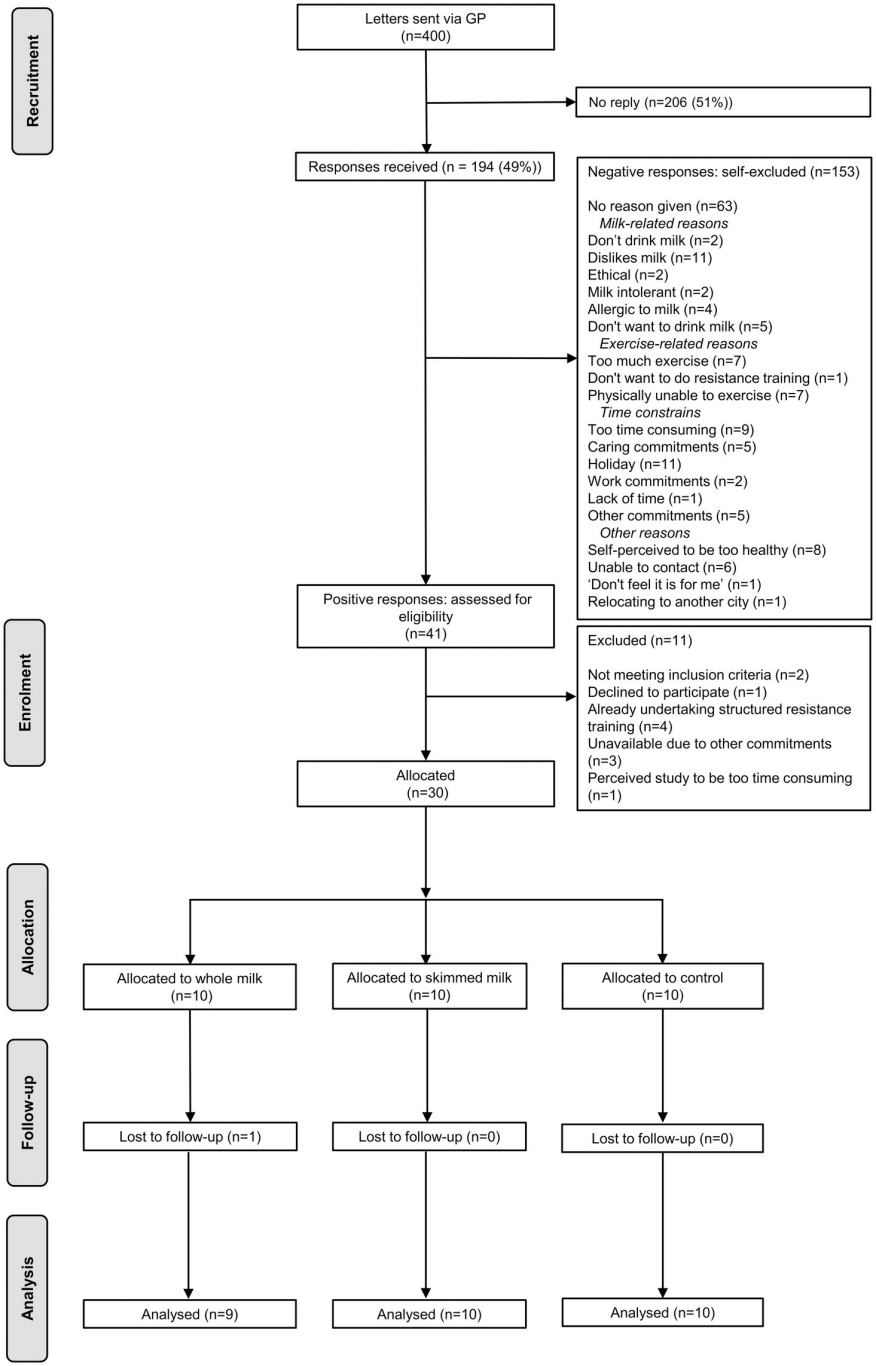
Participant flow diagram. Four hundred letters and participants’ information packs were sent through general practitioner (GP) surgeries within the National Institute for Health Research (NIHR) North East and North Cumbria Clinical Research Network (CRN). Of 194 (49%) replies, 153 participants were negative responders (i.e. self-excluded for a range of reasons), and 41 positive responders were assessed for eligibility against inclusion / exclusion criteria. Thirty participants (18 men and 12 women) were randomised into three study groups, and 29 completed the study.

Community-dwelling older adults aged at least 65 years old were recruited via two primary care practices within the National Institute for Health Research (NIHR) North East and North Cumbria Clinical Research Network (CRN), UK from November 2018 to until April 2019. We used two-step pre-screening approach. First, prospective participants were pre-screened by primary care practices from their patient databases using inclusion and exclusion criteria outlined in the study protocol [22] (e.g. exclusion: any major metabolic, respiratory, cardiovascular conditions, and mental and physical impairments that would preclude safe participation in the intervention; hip and knee replacement; BMI ≥30 kg/m^2^). Prospective participants were mailed a participant information pack inviting them to take part in the study. Second, those responding positively were further pre-screened for eligibility using the SARC-F tool [26] over the phone by a trained research team (CH (exercise physiologist), and LD (health psychologist)) (Fig 1). The SARC-F assesses any difficulties with day-to-day activities (i.e. lifting and carrying 10 pounds, walking across a room, transferring from a chair or bed, and climbing a flight of 10 stairs) and occurrence of falls in the past year [26]. Their eligibility was confirmed in a home screening visit by the research team (CH, LD), and included the assessment of muscle strength (grip strength, GS) and function (gait speed) based on established cut-offs (i.e. <20 kg (women), and <30 kg (men) for low GS; <0.8 m/s or 5 s over 4 m distance for low walking speed) [27]. Briefly, isometric GS was measured using a Jamar^®^ Hydraulic Hand Dynamometer (Model J00105; Anatomy Supply Partners, Atlanta, GA, USA) in a sitting position with the elbow in 90° flexion supported by a fixed arm rest. Participants were instructed to squeeze the dynamometer as hard as possible to assess the maximal force for each hand. Three measurements for each hand alternating between the hands were recorded to the nearest kg. The maximum value of six trials was used to determine GS cut-off (low versus high) to minimise participants to the study groups. For gait speed, a safe and straight 4 m distance was measured and marked in participants’ home by the research team. Participants were instructed to walk between the marks at their usual pace, and their times were recorded with a stop watch.

For invited individuals who responded negatively, the reasons for non-participation (self-exclusion) were recorded (Fig 1). Full details of the recruitment strategy and eligibility criteria can be found in the study protocol [22].

Eligible participants, evaluated by the research team (CH, LD) to be able to participate safely, were allocated to one of the three experimental groups using a minimisation computer programme (MimimPy v0.3 software) [28]. This method aims to balance groups at baseline across several prognostic factors even when sample sizes are small [29], and, in this investigation, minimising was performed using sex and GS. As the target number of participants with low GS was not defined a priori, participants were recruited irrespective of muscle strength and function for whom it was considered safe and potentially beneficial to participate.

### Intervention

#### Resistance exercise programme

The machine-based (Precor Inc., Woodinville, WA, USA) RE programme involved 12 exercise sessions of upper- and lower-body resistance exercise delivered twice-weekly, over 6 weeks. The initial session began with extensive explanation and demonstration of correct lifting technique before participants were familiarised with each exercise (leg press, leg curl, chest press, upright row). Following this, participants’ one-repetition maximum (1RM) was estimated for each exercise using the equation of Brzycki [30,31]. Participants were initially asked to perform repetitions using a load that was perceived to be challenging but manageable before load was progressively increased until momentary failure occurred within 10 repetitions. Estimated 1RM was then calculated based on load lifted and number of repetitions performed.

The remaining exercise sessions comprised a 5-min warm-up (stationary cycling or treadmill walking) followed by 2–3 sets of 8–10 repetitions for each exercise. Training load (kg) was initially set at 70% of estimated 1RM and was increased when participants completed the prescribed number of repetitions in the final set while maintaining proper form in two consecutive workouts. Rest periods of ~60–90 s between sets and ~3 min between exercises were allowed. Exercise sessions were separated by 48 h and were typically ~30 min duration. All RE sessions were delivered at a local authority leisure facility (gym) in groups of 2–4 participants and were supervised by the experienced exercise physiologist (CH).

Training intensity was evaluated using ratings of perceived exertion (RPE) with participants asked to rate how hard and strenuous the physical task felt. Using the CR100^®^ scale [32], participants provided a rating for overall session RPE (sRPE) as well as differential session ratings of perceived exertion for upper-body muscle (sRPE-U) and lower-body muscle exertion (sRPE-L) ~10 minutes after the completion of each RE session [33]. Participants were asked to rate their perception of effort on the scale from 0 to 100, with the encored words describing how difficult the session had felt to them (e.g. 0 ‘not at all’, 35 ‘somewhat hard’, 50 ‘hard’, 75 ‘very hard’, and 100 ‘maximal’). Muscle soreness was evaluated using a Visual Analogue Scale describing the intensity of muscle soreness (e.g. 0 ‘no pain at all’, and 7–10 ‘the worst pain’) at ~6–7 h post-exercise [22]. Blood pressure was measured ~10 min pre and post-exercise (Omron Healthcare Co., Ltd., Muko, Kyoto, Japan).

#### Nutrition intervention

Following each RE session, participants were asked to consume a 500 ml bolus intake of either (1) WM (Arla Cravendale^®^, Arla Foods Ltd., Leeds, UK; 3.6 g fat, 3.4 g protein, and 4.7 g carbohydrate per 100g), (2) SM (Arla Cravendale^®^, Arla Foods Ltd., Leeds, UK; 0.3 g fat, 3.6 g protein, and 4.9 g carbohydrate per 100 g of milk) or (3) isocaloric control drink (cranberry juice, Ocean Spray Classic^®^; The Ocean Spray Manufacturing, Middleboro, MA, USA; 23 kcal/100 g of drink and supplemented with maltodextrin (4 kcal/g) to match whole milk energy content) within ~45 minutes of completing the exercise and prior to leaving the centre. Participants were provided with a further 500 ml dose to consume as part of their usual diet over the next 4–5 hours, and were called by a member of the research team (AG) in the evening to assess their drink compliance, drinking difficulty, and wellbeing.

Participants were asked to maintain their habitual diet and physical activity throughout the intervention period.

### Study outcomes

Primary outcomes were the main focus the study to answer the questions about the feasibility and acceptability of the intervention. Secondary outcomes were linked to the stated aim of providing pilot data to inform future substantive research through exploratory analyses.

#### Primary outcomes

To assess the feasibility and acceptability of the intervention, we investigated the following outcomes: (1) recruitment rate (the number of individuals approached, responding and reasons for not participating in the study); (2) compliance with the intervention (the number of exercise sessions completed, and actual drink consumption divided by possible consumption over the 6-week intervention); (3) attrition (the number of individuals consenting to take part but dropping out without completing a minimum of 10 (83%) exercise sessions, and the reasons why), and (4) adverse health effects, to assess the safety of the intervention.

#### Secondary outcomes: Exploratory pilot data

Secondary outcomes were collected at baseline and post-intervention and included: (1) physical performance (Short Physical Performance Battery (SPPB): balance test, 5-repetition chair stand test, and 4-m gait speed; score 0–12) [34]; (2) muscle strength (the maximum reading of 6 GS measurements (3 for each hand); Jamar^®^ hand-held J00105 dynamometer) [35]; (3) muscle mass (bioelectrical impedance (BIA); TANITA MC-780MA Body Composition Analyzer, TANITA Corporation, Tokyo, Japan) [36], and (4) self-reported QoL (12-Item Short Form Survey 12, SF-12) [37] to calculate physical components summary (PCS) and mental component summary (MCS) scores ranging from 0–100 [38]. The exploration of secondary outcomes will aid the design and refinement of future substantive research.

#### Other measures and participants’ characteristics

To investigate further the compliance with drink consumption, participants were asked to rate their experiences on a Likert scale using the questions assessing: (1) difficulty consuming 2 l milk/control drink a week; (2) amount of drink as a barrier for participation in the study, and (3) amount of drinks affecting appetite. Similarly, participants were asked to rate their overall experience in the study and difficulty in keeping the commitment to the study.

Socio-demographic characteristics included age, sex, marital status, occupational class, years of education, and deprivation index.

Health-related characteristics included self-rated quality of life, number of diseases diagnosed by a doctor (from a list of common metabolic, cardiovascular, cerebrovascular, respiratory, kidney and other conditions), number of prescribed medication, depression (15-item Geriatric Depression Scale, GDS), cognitive health (Mini-Mental State Examination, MMSE; 0–30 score), and disability in activities of daily living (Barthel Index; 0–20 score) [39].

Lifestyle variables included smoking status, alcohol intake, diet, and physical activity (PA). Diet was assessed using 24-h dietary recall (Intake24 online tool; https://intake24.co.uk/) [40] to evaluate whether supplementation with milk or juice affected participants’ diet. Self-reported PA was assessed using a PA questionnaire asking about the type and frequency of PA involved in participants’ daily lives that were either highly energetic, moderately energetic, or mildly energetic. The PA score (0–18) was calculated as described previously [41], and categorised into low (0–1), medium (2–6), and high (7–18) score. Specifically, from the frequency of each activity (i.e. ≥3 times/week (score 3); 1–2 times/week (score 2); 1–2 times/month (score 1), and hardly ever/never (score 0) we created three outcome variables: very energetic, moderately energetic, and mildly energetic activities (score range of 0–3 for each). An overall PA score was derived using the following formula: (3×very energetic activities score) + (2×moderately energetic activities score) + (1×mildly energetic activities score).

Anthropometric measures included height (calculated from DEMI span formula for men and women), waist-hip ratio, and calf circumference (cm). From bioimpedance data, we estimated weight (kg), BMI (underweight <18.0 kg/m^2^; normal 18.5–24.9 kg/m^2^; overweight/obese >25 kg/m^2^), appendicular skeletal muscle mass (ASM; a sum of lean muscle mass in arms and legs), and calculated skeletal muscle index (SMI, kg/m^2^) using Baumgartner et al formula [42]. Sarcopenia status was assessed using the revised European Working Group on Sarcopenia in Older Adults second algorithm (EWGSOP2) [1,2].

All measurements and characteristics were assessed at baseline interview, except diet, self-reported heath, Barthel Index, and bioimpedance data, which were assessed at baseline and post-intervention interview [22]. Questions about compliance with drinks consumption, and participants’ general experience in the study were assessed at post-intervention interview.

### Statistical analysis

Descriptive statistics were reported for all primary outcomes, including percentages (for categorical variables), means and standard deviations (M, SD) (for continuous variables). Descriptive statistics were also used to summarise exploratory pilot data (secondary outcome measures pre- and post-intervention across the intervention groups). We used paired t-test to calculated the point estimates of the sample mean differences ($\bar{\text{x}}$) with 95% confidence intervals (CI) for secondary outcomes in all participants. Statistical analyses were performed using SPSS (v.25; IBM Corp., Armonk, NY, USA).

## Results

The number of participants approached, recruited, randomly assigned, and analysed are presented in the participant flow diagram (Fig 1).

Baseline characteristics of recruited participants are summarised in Table 1. The intervention and control groups had similar sociodemographic, health and lifestyle characteristics.

**Table 1:** Characteristics of participants randomised to the intervention at baseline.

Characteristics	All participants	Whole milk	Skimmed milk	Control
n	30	10	10	10
***Socio-demographic***				
Age (M, SD)	71.7 (3.6)	72.0 (2.7)	72.2 (4.1)	70.8 (4.0)
Sex (men/women, %)	18/12 (40)	6/4	6/4	6/4
Marital status n (%)				
Married	19 (63.3)	4 (40)	7 (70)	8 (80)
Not married	11 (36.7)	6 (60)	3 (30)	2 (20)
Occupational class* n (%)				
Higher managerial/administrative	13 (43.3)	5 (50)	5 (50)	3 (30)
Intermediate occupations	15 (50)	5 (50)	4 (40)	6 (60)
Routine/manual occupations	1 (3.3)	0 (0)	0 (0)	1 (10)
Never worked	1 (3.3)	0 (0)	1 (10)	0 (0)
Index of Multiple Deprivation^†^				
Poor areas (<25^th^ percent)	8 (26.7)	5 (50)	2 (20)	1 (10)
Intermediate (25-75^th^ percent)	15 (50)	5 (50)	3 (30)	7 (70)
Affluent areas (>75^th^ percent)	7 (23.3)	0 (0)	5 (50)	2 (20)
Total years of education				
0–9	0 (0)	0 (0)	0 (0)	0 (0)
10–11	18 (60)	6 (60)	7 (70)	5 (50)
≥12	12 (40)	4 (40)	3 (30)	5 (50)
***Health status***				
Self-rated general health n (%)				
Excellent/ very good	17 (56.7)	5 (50)	7 (70)	5 (50)
Good	9 (30)	4 (40)	2 (20)	3 (30)
Fair/poor	4 (13.3)	1 (10)	1 (10)	2 (20)
Number of chronic disease n (%)				
0	3 (10)	1 (10)	1 (10)	1 (10)
1	17 (56.7)	7 (70)	5 (50)	5 (50)
2–3	10 (33.3)	2 (20)	4 (40)	4 (40)
Polypharmacy n (%)				
0–4 medications	20 (66.7)	8 (80)	5 (50)	7 (70)
≥5 medications	10 (33.3)	2 (20)	5 (50)	3 (30)
MMSE (M, SD)	29.4 (1.1)	29 (1.6)	29.6 (1.0)	29.6 (0.5)
Barthel Index (M, SD)	19.9 (0.4)	19.9 (0.3)	19.8 (0.6)	20 (0)
GDS (M, SD)	1.1 (1.8)	0.40 (0.7)	1.6 (2.8)	1.3 (1.2)
Sarcopenia status, EWGSOP2^‡^				
No sarcopenia	28 (93.3)	10 (100)	9 (90)	9 (90)
Probable sarcopenia	2 (6.7)	0 (0)	1 (10)	1 (10)
***Lifestyle***				
Smoking status n (%)				
Never smoker	13 (43.3)	4 (40)	5 (50)	4 (40)
Former smoker	12 (40)	3 (30)	4 (40)	5 (50)
Current smoker	5 (16.7)	3 (30)	1 (10)	1 (10)
Current alcohol intake n (%)				
0–2 units	12 (40)	4 (40)	4 (40)	4 (40)
3–4 units	2 (6.7)	0 (0)	1 (10)	1 (10)
5 and more units	16 (53.3)	6 (60)	5 (50)	5 (50)
Physical activity^¶^ n (%)				
Low (score 0–1)	0 (0)	0 (0)	0 (0)	0 (0)
Medium (score 2–6)	4 (13.3)	2 (20)	0 (0)	2 (20)
High (score 7–18)	26 (86.7)	8 (80)	10 (100)	8 (80)
Protein g/kg BW/day (M, SD)^5^	0.9 (0.3)	0.98 (0.4)	0.84 (0.3)	0.88 (0.3)
Energy, MJ (M, SD) ^#^	6.18 (2.1)	6.78 (2.1)	5.35 (1.7)	6.41 (2.5)
***Anthropometry***				
Waist-hip ratio (M, SD)	0.9 (0.1)	0.9 (0.1)	0.9 (0.1)	0.9 (0.1)
Calf circumference, cm (M, SD)	31.2 (4.1)	31.1 (4.4)	31.9 (4.4)	30.7 (3.9)
BMI, kg/m^2^ M (SD) ^§^	25.8 (2.9)	24.91 (2.9)	25.5 (2.6)	27 (3)
BMI categories n (%)				
Underweight (<18.5)	0 (0)	0 (0)	0 (0)	0 (0)
Normal (18.5–24.9)	12 (40)	4 (40)	5 (50)	3 (30)
Overweight (>25.0)	18 (60)	6 (60)	5 (50)	7 (70)

*Based on the National Statistics Socio-Economic Classification System, The Office of National Statistics, UK.

^†^Levels of poverty in current area of residency; higher scores indicating less deprivation, and categorised in quartiles, and Q2 and Q3 combined to form ‘intermediate group’.

^‡^Based on baseline values and EWGSOP2 definition [1,2].

^¶^Based on a physical activity questionnaire about the type and frequency of highly energetic, moderately energetic, and mildly energetic in participants’ daily life, and calculated as described previously [40].

^#^Based on Intake24 and food composition codes and their energy and nutritional content [39].

^§^Estimated from bioimpedance and height calculated from DEMI span measurements.

Abbreviations: EWGSOP2, European Working Group on Sarcopenia in Older People; GDS, Geriatric Depression Scale; M, mean; MMSE, Mini Mental State Examination; SD, standard deviation.

### Primary outcomes

#### Recruitment

The response rate was 49% (194 responses received out of 400 invitation packs sent from November 2018 to February 2019), and the recruitment rate was 73.2% (30 participants recruited out of 41 participants screened for eligibility and randomised to the study) (Fig 1). Specifically, 153 (78.9%) out of 194 replies were negative responders who self-excluded for the reasons related to milk (17%), exercise (9.8%), time constrains (21.6%), other reasons (10.5%), or no reasons given (41.2%). Twenty-one percent (n = 41) were positive responders who were assessed for eligibility (Fig 1).

#### Compliance, attrition and safety

Twenty-nine participants (96.7%) completed the intervention (March—May 2019) with an overall attendance of 97.1% (338 out of a possible 348 sessions) (Table 1). One participant in the WM+RE group withdrew from the study during the intervention period because of a previously sustained musculoskeletal injury unrelated to the study. Twenty-two participants completed all exercise sessions (75.9%), 4 participants completed 11 sessions (13.8%) and 3 participants completed 10 sessions (10.3%). Reasons for missed sessions were: illness (4 sessions), holiday (4 sessions), childcare commitments (1 session) and medical appointment (1 session). No adverse heath events were reported throughout the duration of the study.

#### Compliance with resistance exercise programme and nutrition intervention

Training intensity for each exercise and participants rating of perceived exertion are presented in Table 2. Mean RPE values across the groups indicated that participants rated exercise intensity as between ‘somewhat hard’ and ‘hard’. The compliance rate with drink consumption was 97.1± 5.9% (WM+RE), 98.3±3.5% (SM+RE) and 95±7.3% (C+RE). Data describing muscle soreness, change in appetite because of milk/control drink consumption, and participants’ overall experience in the study across the intervention groups are presented in Table 2.

**Table 2:** Feasibility, acceptability and general experience in participants completing the study.

Intervention characteristics	All participants	Whole milk	Skimmed milk	Control
Completed the study, n (%)	29 (96.7)	9 (90)	10 (100)	10 (100)
Number of sessions completed, M (SD)	11.7 (0.7)	11.7 (0.7)	11.8 (0.4)	11.5 (0.9)
Absolute % (SD)	97.1 (5.6)	97.2 (5.9)	98.3 (3.5)	95.8 (7.1)
***Mean training intensity %1RM*, *M (SD****)*				
Leg press	73.7 (3.5)	73.4 (1.6)	74.1 (1.9)	74.9 (1.9)
Leg curls	72.4 (3.6)	73.6 (3.3)	72.2 (2.2)	72.8 (1.6)
Chest press	72.9 (5.5)	74.2 (2.9)	74.1 (4.6)	71.9 (6.3)
Upper row	72.4 (3.8)	71.4 (1.0)	72.5 (1.7)	74.5 (3.2)
Ratings of Perceived Exertion AU*, ***M (SD)***				
Overall (sRPE)	38.4 (19)	38.6 (21.3)	35.1 (18.2)	41.7 (19.3)
Upper-body (sRPE-U)	42.5 (21)	35.3 (19.7)	34.5 (18.2)	37.1 (20.2)
Lower Body (sRPE-L)	35.7 (18.7)	44.8 (24.8)	36.4 (18.8)	46.6 (20.1)
*Muscle soreness*^†^, *M (SD)*				
6 hours post-RE	0.2 (0.4)	0.2 (0.3)	0.4 (0.6)	0.1 (0.1)
***Milk/control drink consumption/compliance (total)*** ^‡^				
Absolute % (SD)	96.8 (5.7)	97.1 (5.9)	98.3 (3.5)	95 (7.3)
Difficulty consuming 2 l/week, n (%)				
Easy or very easy	27 (93.1)	9 (100)	8 (80)	10 (100)
Neutral	2 (6.9)	0 (0)	2 (20)	0 (0)
Difficult or very difficult	0 (0)	0 (0)	0 (0)	0 (0)
Amount of drink a barrier, n (%)				
Not at all	27 (93.1)	9 (100)	8 (80)	10 (100)
Somewhat	2 (6.9)	0 (0)	2 (20)	0 (0)
Moderate	0 (0)	0 (0)	0 (0)	0 (0)
Extreme	0 (0)	0 (0)	0 (0)	0 (0)
Change in diet^¶^, n (%)				
No affect	25 (86.2)	8 (88.9)	9 (90)	8 (80)
Minor affect or neutral	3 (33.3)	1 (11.1)	1 (10)	1 (10)
Moderate or major	1 (3.4)	0 (0)	0 (0)	1 (10)
***General experience*, *n (%)***				
Overall study experience				
Excellent or very good	26 (89.7)	9 (100)	9 (100)	8 (80)
Good	3 (10.3)	0 (0)	1 (10)	2 (20)
Poor or fair	0 (0)	0 (0)	0 (0)	0 (0)
Keeping commitment to the study				
Easy or very easy	27 (93.1)	9 (100)	10 (100)	8 (80)
Neutral	2 (6.9)	0 (0)	0 (0)	2 (20)
Difficult or very difficult	0 (0)	0 (0)	0 (0)	0 (0)

*AU: 0, nothing at all; 12, easy; 22, moderate; 35, somewhat hard; 50, hard; 70, very hard; 100 maximal.

^†^Muscle soreness scale 0–10 AU; 0, no pain; 10, worse pain.

^‡^Maximum 6000 ml immediately post-RE and 6000 ml at home over the 6-week intervention.

^¶^Change in diet because of drinking amount whilst in the study.

Abbreviations: 1RM, one repetition maximum; AU, arbitrary units; M, mean; RPE, ratings of perceived exertion, SD, standard deviation.

### Secondary outcomes: Exploratory pilot data

Collection of exploratory pilot data (secondary outcomes) to inform future trials was successful and completed as outlined in the study protocol [22].

Baseline and post-intervention values for muscle strength, mass, physical performance, and QoL outcomes in all participants are presented in Table 3. Differences (mean delta scores, Δ) in GS, 4-m gait speed, 5-chair rises, and PCS score of SF-12 (QoL scale) pre- and post-intervention in those completing the study (n = 29) are presented in S1 Table and S1 Fig. Exploratory analysis demonstrated the following mean Δ: 0.9±3.4 kg in GS, 1.8±2.2 s in 5-chair rises, 0.1±0.1 m/s in gait speed, and 1.2±4.6 points in the QoL physical components score, with no differences between the groups in participants completing the intervention. These slight improvements were likely due to RE, practice effect and measurement bias.

**Table 3:** Unadjusted values for measures of muscle strength, mass, physical performance, and quality of life in all participants and across the groups at baseline and post-intervention: Exploratory pilot data.

	Baseline				Post-intervention				
	All participants	Whole milk	Skimmed milk	Control	All participants	Whole milk	Skimmed milk	Control	$\bar{\text{x}}$; 95% CI^†^
n	30	10	10	10	29	9	10	10	
***Muscle strength***									
Grip strength (kg), M (SD)	30.9 (10)	32.9 (11.9)	28.3 (7.9)	31.6 (10.2)	31.6 (10.7)	32.8 (13.2)	29.3 (7.5)	32.9 (11.6)	0.9; -0.3 to 2.2
Muscle mass*									
ASM kg, M (SD)	20 (4.2)	20.2 (4.2)	19.3 (4.3)	20.5 (4.6)	20.2 (4.4)	20.5 (4.6)	19.5 (4.6)	20.5 (4.4)	0.1; -0.1 to 0.3
SMI kg/m^2^, M (SD)	7.2 (1.1)	7.2 (1.1)	7.1 (1.2)	7.4 (1)	7.3 (1.1)	7.3 (1.2)	7.2 (1.3)	7.4 (1)	0.1; 0 to 0.3
***Physical performance***									
SPPB score, M (SD)	11.1 (1.2)	11.2 (1.3)	11.0 (1.2)	11.1 (1.1)	11.8 (0.7)	11.7 (0.7)	11.7 (1)	11.9 (0.3)	0.6; 0.2 to 0.9
SPPB categories, n (%)									
Low (score 0–6)	0 (0)	0 (0)	0 (0)	0 (0)	0 (0)	0 (0)	0 (0)	0 (0)	NA
Intermediate (score 7–9)	4 (13.3)	1 (10)	2 (20)	1 (10)	1 (3.4)	0 (0)	1 (10)	0 (0)	NA
High (score 10–12)	26 (86.7)	9 (90)	8 (80)	9 (90)	28 (96.6)	9 (100)	9 (90)	10 (100)	NA
Side-by-side stand (s), M (SD)	10 (0.0)	10.0 (0.0)	10.0 (0.0)	10.0 (0.0)	10.0 (0.0)	10.0 (0.0)	10.0 (0.0)	10.0 (0.0)	0
Semi-tandem stand (s), M (SD)	10 (0.0)	10.0 (0.0)	10.0 (0.0)	10.0 (0.0)	10.0 (0.0)	10.0 (0.0)	10.0 (0.0)	10.0 (0.0)	0
Tandem stand (s), M (SD)	8.4 (3)	8.7 (2.8)	8.7 (2.8)	7.8 (3.7)	9.8 (1.2)	9.3 (2.2)	10.0 (0.0)	10 (0.2)	1.1; 0.1 to 2.3
5-chair rises (s), M (SD)	10.6 (2.6)	10.4 (2.4)	11.1 (3.1)	10.3 (2.5)	8.7 (2.2)	9 (2.7)	8.9 (2.3)	8.2 (1.7)	1.8; 1 to 2.6
4-m gait speed (s), M (SD)	3.4 (0.7)	3.3 (0.6)	3.6 (0.8)	3.4 (0.6)	3.2 (0.6)	3.2 (0.5)	3.3 (0.7)	3 (0.5)	0.2; 0.1 to 0.3
Gait speed (m/s), M (SD)	1.2 (0.2)	1.3 (0.2)	1.2 (0.2)	1.2 (0.2)	1.3 (0.2)	1.3 (0.2)	1.3 (0.3)	1.4 (0.2)	0.1; 0 to 0.1
***Quality of Life***^‡^									
MCS of SF12, M (SD)	57.8 (3.6)	58.5 (1.9)	57.1 (5.5)	57.7 (2.6)	58.2 (3.7)	58.8 (2.1)	58.2 (5.7)	57.6 (2.5)	0.4; -0.8 to 1.7
PCS of SF12, M (SD)	51 (6.9)	52.6 (4.2)	50.5 (10)	50 (5.6)	52.4 (5.7)	54.2 (2.8)	50.8 (9)	52.5 (2.8)	1.2; -0.6 to 3

*Based on bioimpedance data. SMI calculated using Baumgartner et al [41] formula.

^† $\bar{\text{x}}$^ (mean difference) and 95% CI (confidence interval) for the difference in continuous measures pre- and post-intervention (all participants).

^‡^Score range 0–100 for each scale; calculation based on Ware et al [40].

Abbreviations: ASM, appendicular skeletal muscle mass; M, mean; MCS, mental component score; NA, not applicable; PCS, physical components core; SD, standard deviation; SMI, skeletal muscle index; SPPB, Short Physical Performance Battery.

## Discussion

The main finding of this pilot study was that a combined RE programme and whole food intervention (milk) delivered in a community setting twice-weekly for 6 consecutive weeks was feasible and acceptable to community-dwelling older adults aged ≥65 years. The study was successful in collecting exploratory pilot data to inform the design of future substantive research. To our knowledge, this is the first study to use milk as a whole food approach in combination with RE and assess the feasibility and acceptability of such an intervention in older adults living in the community.

The present study had ~50% response rate (achieved though the involvement of primary care and screening of patient databases), with a low drop-out rate. Difficulties with recruiting older adults to interventions and higher dropout rates have been widely acknowledged, especially in vulnerable older adult populations [43–45]. Intrinsic health beliefs, influence of significant others, and encouragement from medical professionals have been identified as motivators for older adults with and without sarcopenia and frailty to participate and adhere to an exercise and/or nutrition intervention [45].

Comparison of our findings for the primary aims (e.g. attendance rate, compliance, attrition) with other studies that used whole food combined with exercise interventions for sarcopenia in older adults is limited because of differences in intervention and delivery characteristics between studies. Such differences include intervention duration, frequency, setting, sample size, and the type and amount of whole food consumed across the studies. Typically, supervised interventions (e.g. exercise trainers) and frequent contacts with participants are reported to achieve higher attendance and compliance rates [46]. Despite this, however, our attendance and compliance figures compare favourably.

For example, the mean attendance rate of Japanese older adults aged 65–78 (n = 56) in a twice-weekly supervised exercise programme combined with 250 ml fortified milk over 12 weeks was 70%, with the mean percentage milk intake of 95% [47]. Similarly, in a 4-month study of sarcopenic men aged 60–75 years (n = 30) from Quebec, the average compliance to the supervised RE programme with 375 ml fortified milk was 90% [48]. In a study of Australian women aged 60–90 years (n = 100) residing in retirement villages, unsupervised consumption of ~160 g lean meat after supervised RE twice-weekly was 81% [49].

MIlkMAN study involved a supervised and resource intensive intervention (i.e. supervised training and milk intake; phone calls post-intervention), which led to high attendance and compliance but raises questions about wider translation and long-term adherence when the support is not in place. A 12-week supervised RE intervention conducted in Belgium in a local gym 3 times a week with community-dwelling older adults aged 60–80 years observed 85% training adherence and high levels of self-determined motivation, but relatively modest long-term adherence after the completion of the supervised training [50]. Whilst supervised exercise may have limited positive effects on motivation for continued exercise engagement in older adults, future studies are needed to develop strategies aimed at identifying motivators and overcoming barriers in older adults to adhere to exercise programme without supervision.

Equally, exercise and nutrition programme preferences of older adults have to be considered in future trials to increase recruitment and adherence to interventions (e.g. preferred type and location, health beliefs, peer support, medical encouragement) [45]. Hence, this pilot study evaluated the feasibility and acceptability of a community-based intervention that involved the consumption of 1 l of milk to achieve ~40 g protein intake post-exercise. This data will be used to inform the design of a future substantive trial.

### Strengths and limitations

This pilot study has several strengths, including the fulfilment of the study primary aims, achieved by engaging an expert multidisciplinary research team (health psychologist, exercise physiologist, clinician on call), high level interaction with participants, and no recorded adverse events. The study was successful in collecting exploratory pilot data (secondary outcomes) for future substantive research. However, it is acknowledged that the limited sample size and short intervention duration (twice-weekly over 6 weeks) meant that an assessment of safety and intervention effect on muscle function was limited.

The study has several limitations mostly pertaining to recruitment of the target population, sample size and assessments used in the study, which were discussed in detail in the study protocol [22]. Briefly, to reach the recruitment targets within the proposed timeframe, the study also included participants with no difficulties with day-to-day activities (assessed by SARC-F), and having normal GS and gait speed. Specifically, only 20% (6 out of 30 recruited participants) had low GS (<20kg in women, and <30kg in men), and 6.7% (2/30) had low gait speed (<0.8m/s) at screening interview (details not shown), suggesting that some participants in the study may have a risk of sarcopenia in the future. Thus, the sample was not representative of people with sarcopenia in the population because it included participants with relatively healthy muscle. In addition, participants had protein intake >0.8/kg BW/day at baseline and high self-reported physical activity. They were also well educated, more likely to belong to higher occupational/social class, and less likely to live in poor areas, which may have affected their lifestyle choices, including diet and exercise. Previous studies have shown no additional anabolic effect of milk or extra protein supplements above those of RE in relatively healthy, active and well-nourished older adults [51,52]. Furthermore, future substantive research should include a measure of overall diet quality and detailed estimation of participants’ micro- and macronutrient for sample characterisation and multivariable analyses examining the effect of nutrition intervention.

Changes in the recruitment strategy are needed to increase the potential to recruit individuals with muscle weakness and low protein intake to a larger trial [43]. There were limitations to assessments of muscle strength (GS), mass (BIA), and physical performance (SPPB) used in the study. For example, physical performance at baseline was high (i.e. SPPB >11points), indicating ceiling effect and test limits to detect change in function. Similar ceiling effect could be applied to GS (i.e. only 20% had low GS at screening interview). Additionally, a future definitive trial will include repeat assessment of 1RM via submaximal testing at baseline and post-intervention, which will allow for a more reliable and internally valid assessment of muscle strength, and for a more specific detection of muscle strength changes, including clinically meaningful change [53].

Limitations to the BIA method for estimating lean body mass in older adults should be considered for the future research, including low sensitivity to detect changes in muscle mass, and the effect of hydration/dehydration and physical activity on the measurements [54], the latter being not considered in this pilot. Although the study successfully collected exploratory pilot data (secondary outcomes), observed delta scores were likely related to exercise, whilst practice effect and bias in measurements could not be excluded.

### Implications for future work

Several implications for the future work should be considered, including those related to (1) recruitment (i.e. screening of the target population (older adults at risk of sarcopenia) via multiple GP surgeries, screening tool for a rapid detection of individuals at risk and appropriate cut-offs, exclusion/inclusion criteria to maximise the recruitment); (2) sample size; (3) randomisation (i.e. criteria to balance the study arms); (4) intervention (i.e. duration, choice of exercise stimulus, exercise delivery, analysis of macronutrient content in milk); (5) details for primary outcomes (i.e. assessments, effect size, definition of clinically meaningful change in muscle function), (6) in-depth collection of qualitative evidence on the motivators and barriers for community-based interventions and continued engagement after the study completion, and (7) process evaluation for intervention studies.

For example, there may be more benefit from protein supplementation after exercise in individuals with physical frailty [55] and malnutrition [56]. Randomising such individuals to a longer duration trial (e.g. 10–12 weeks, repeated blocks of 6-week intervention) would help to establish clinically meaningful change and differences across the arms in measures of muscle function that may have practical implications. Although RE has been reported to clinically improve muscle quality and function in older adults even after 6 weeks [57], the evidence for positive effects of protein-rich foods above those provided by RE in healthy and active older adults has been inconclusive [49,51]. MIlkMAN pilot study succeeded to recruit participants with relatively heathy muscle (i.e. only 8 had either low GS or slow gait speed). Recruiting individuals with reduced muscle function and probable sarcopenia into clinical trials has been challenging for several reasons, including the diversity of muscle-related outcomes in assessing the complexity of muscle heath, and the absence of routine diagnostic of sarcopenia in clinical practice [1,2,5,6,11]. Recent revision of sarcopenia definition and recommendations for making a diagnosis in practice [1,2] will aid the inclusion of sarcopenia into primary care, which already routinely collects an electronic frailty score (Frailty Index, eFI) [58] across the UK. Thus wider use of GP surgeries for recruitment and available pre-screening tools (SARC-F, eFI) may increase the potential for targeting older adults who are most likely to benefit from the nutrition and exercise intervention. For the process evaluation in a future trial, a mixed-methods approach may be used to understand, for example, the contextual factors which may either weaken or strengthen the effect of the intervention (i.e. factors external to the intervention such as participants’ circumstances, attitudes, social norms and resources) [59].

## Conclusions

This community-based milk and RE intervention was feasible and acceptable to older adults. The study successfully collected exploratory pilot data to inform the design of future substantive research. However, more consideration is needed to recruit older adults who may benefit from this approach the most for inducing positive changes in muscle health and performance.

## Supporting information

S1 Checklist CONSORT 2010 checklist of information to include when reporting a pilot or feasibility trial*. (DOC)

S1 Fig Unadjusted pre- and post-intervention values in selected outcomes in participants completing the study and across the groups. (DOCX)

S1 Table Unadjusted pre- and post-intervention values in selected outcomes in participants completing the study and across the groups. (DOCX)

S1 File (DOCX)
