# Supplementary material for: Feasibility and acceptability of a milk and resistance exercise intervention to improve muscle function in community-dwelling older adults (MIlkMAN): Pilot study
Source: PLoS One. 2020 Jul 10;15(7):e0235952. doi: 10.1371/journal.pone.0235952 (PMC7351162; doi:10.1371/journal.pone.0235952)

**S1 Fig. Unadjusted pre- and post-intervention values in selected outcomes in participants completing the study and across the groups.** A-D. Mean delta scores (Δ) with standard deviation (SD) in grip strength (kg), time to complete 4 m gait speed (m/s), 5-chair rises (s), and physical performance summary (PCS) of the 12-Item Short Form Survey (SF-12) in participants who completed the study (n=29). No differences between the three groups were observed. Error bars represent SEM (standard error of the mean).


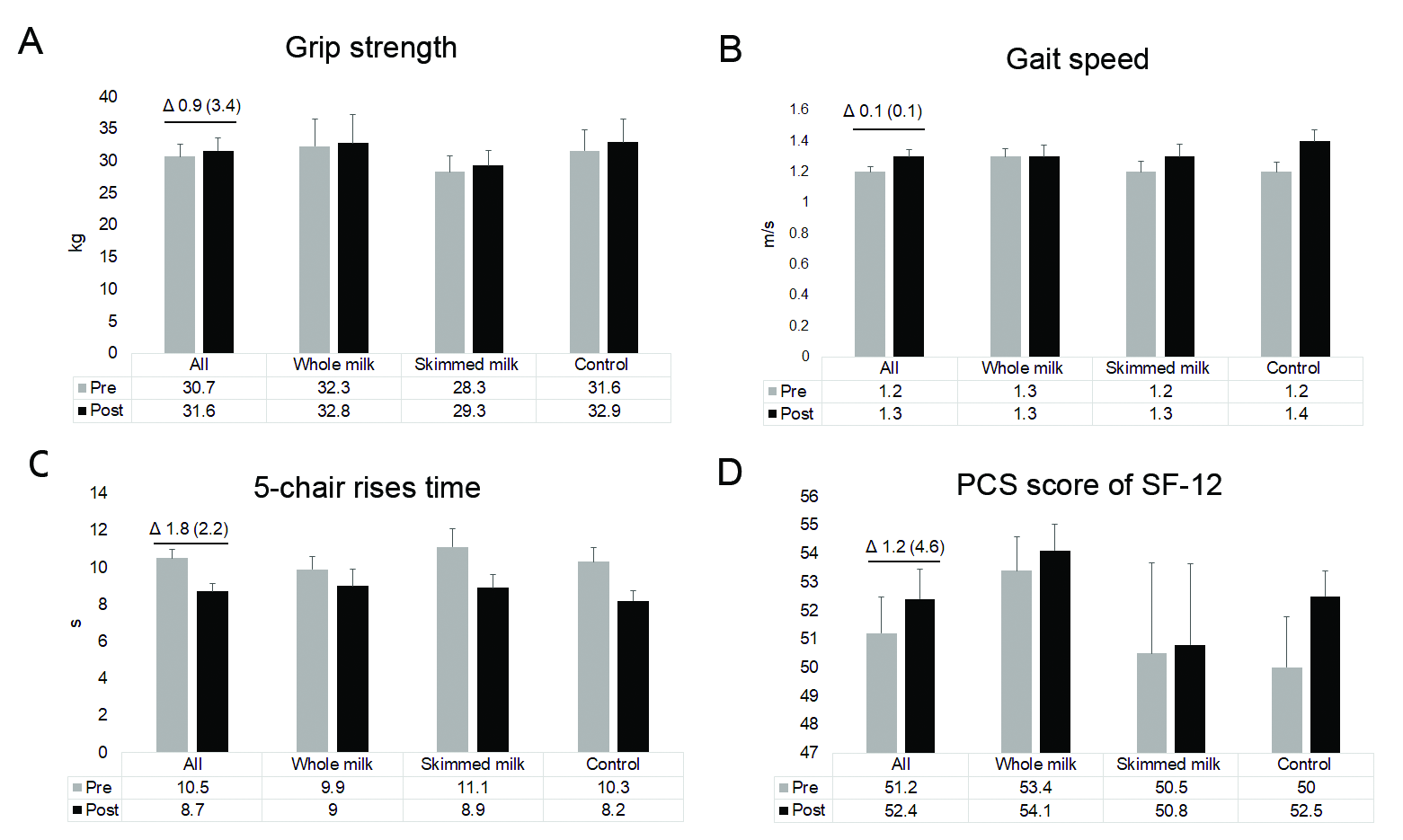

Supplement: S1 Fig — (DOCX) [file pone.0235952.s002.docx]
