# Supplementary material for: Feasibility and acceptability of a milk and resistance exercise intervention to improve muscle function in community-dwelling older adults (MIlkMAN): Pilot study
Source: PLoS One. 2020 Jul 10;15(7):e0235952. doi: 10.1371/journal.pone.0235952 (PMC7351162; doi:10.1371/journal.pone.0235952)
Supplement: S1 Table — (DOCX) [file pone.0235952.s003.docx]

**S1 Table. Unadjusted pre- and post-intervention values in selected outcomes in participants completing the study and across the groups.**

| **Outcome** | **All participants** | **Whole milk** | **Skimmed milk** | **Control** |
| --- | --- | --- | --- | --- |
| ***Grip strength, kg (SEM)*** |  |  |  |  |
| Pre-intervention | 30.7 (1.88) | 32.3 (4.19) | 28.3 (2.51) | 31.6 (3.24) |
| Post-intervention | 31.6 (1.99) | 32.8 (4.41) | 29.3 (2.39) | 32.9 (3.68) |
| **Gait speed, m/s (SEM)** |  |  |  |  |
| Pre-intervention | 1.2 (0.04) | 1.3 (0.05) | 1.2 (0.07) | 1.2 (0.06) |
| Post-intervention | 1.3 (0.04) | 1.3 (0.07) | 1.3 (0.08) | 1.4 (0.07) |
| **5-chair rises, s (SEM)** |  |  |  |  |
| Pre-intervention | 10.5 (0.48) | 9.9 (0.69) | 11.1 (0.97) | 10.3 (0.79) |
| Post-intervention | 8.7 (0.41) | 9 (0.91) | 8.9 (0.73) | 8.2 (0.53) |
| **PCS score of SF-12, M (SEM)** |  |  |  |  |
| Pre-intervention | 51.2 (1.29) | 53.4 (1.18) | 50.5 (3.17) | 50 (1.77) |
| Post-intervention | 52.4 (1.06) | 54.1 (0.93) | 50.8 (2.84) | 52.5 (0.89) |

Abbreviations: M, mean; PCS, physical components core of SF-12 questionnaire; SEM, standard error of the mean.
