# Supplementary material for: Feasibility and acceptability of a milk and resistance exercise intervention to improve muscle function in community-dwelling older adults (MIlkMAN): Pilot study
Source: PLoS One. 2020 Jul 10;15(7):e0235952. doi: 10.1371/journal.pone.0235952 (PMC7351162; doi:10.1371/journal.pone.0235952)
Supplement: S1 File — (DOCX) [file pone.0235952.s004.docx]

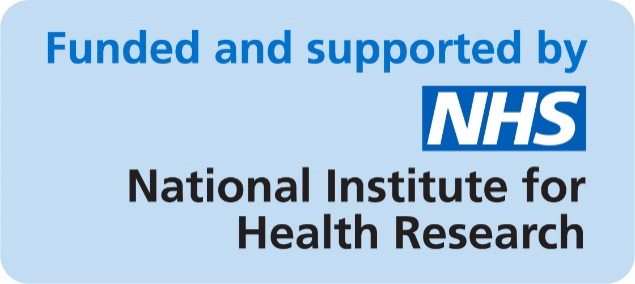


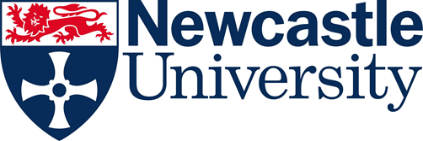

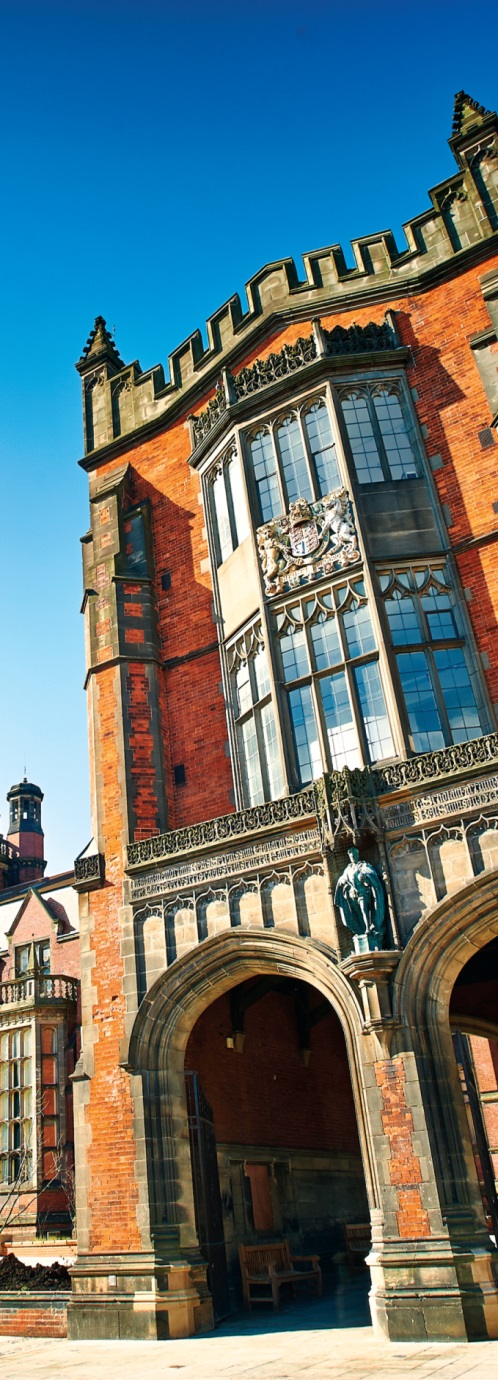


**STUDY PROTOCOL**

***Full name*: MIlk Intervention Muscle AgeiNg (MIlkMAN): pilot**

***Short name*: MIlkMAN**

**
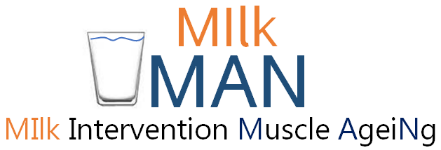
**

Chief Investigator

Dr Terry Aspray

Honorary Clinical Senior Lecturer

Institute of Cellular Medicine, Newcastle University; Bone Clinic, Freeman Hospital, Newcastle

*Contact*: Email: [terry.aspray@newcastle.ac.uk](mailto:terry.aspray@newcastle.ac.uk)

*Main role*: Overall responsibility of the research and scientific support of the study.

Principal Investigator (main study contact)

Dr Antoneta Granic

Interdisciplinary AGE Research Fellow

Institute of Neuroscience; NIHR Newcastle Biomedical Research Centre and Newcastle upon Tyne Hospitals NHS Foundation Trust; Newcastle Institute for Ageing, Newcastle University

*Contact*: Email: [antoneta.granic@newcastle.ac.uk](mailto:antoneta.granic@newcastle.ac.uk)

Telephone 01912481112

Fax 01912481101

*Main role*: Responsible for the management of the study, study protocol and reports, recruitment and consents, and overseeing research activity/assessment with participants. Overall responsibility for the study.

# Co-investigators

Dr Karen Davies

Resource Manager, Ageing Geriatrics & Epidemiology (AGE) Research Group

Institute of Neuroscience; NIHR Newcastle Biomedical Research Centre and Newcastle upon Tyne Hospitals NHS Foundation Trust; Newcastle Institute for Ageing, Newcastle University

*Contact*: Email: [karen.davies@newcastle.ac.uk](mailto:karen.davies@newcastle.ac.uk)

*Main role*: Overseeing the management of the study, study protocols and reports, recruitment, and research activities with participants.

Prof Emma Stevenson

Professor of Sport and Exercise Science

Institute of Cellular Medicine, Newcastle University

*Contact*: Email: [emma.stevenson@newcastle.ac.uk](mailto:emma.stevenson@newcastle.ac.uk)

*Main role*: Responsible for all aspects of nutritional and exercise intervention programme and scientific support of the study.

Professor Avan A. Sayer

Director, NIHR Newcastle Biomedical Research Centre & Professor of Geriatric Medicine

Institute of Neuroscience; NIHR Newcastle Biomedical Research Centre and Newcastle upon Tyne Hospitals NHS Foundation Trust; Newcastle Institute for Ageing, Newcastle University

*Contact*: Email: [avan.sayer@newcastle.ac.uk](mailto:avan.sayer@newcastle.ac.uk).

*Main role*: Academic lead and scientific support of the study.

# Sponsor

Northumbria Healthcare NHS Foundation Trust,

Research and Development, North Tyneside General Hospital

North Shields,

Newcastle upon Tyne,

NE29 8NH

*Contact*: Peta Heslop, [Peta.Heslop@northumbria-healthcare.nhs.uk](mailto:Peta.Heslop@northumbria-healthcare.nhs.uk);

# Funder

NIHR Newcastle Biomedical Research Centre, £91,041.18 for 14 to 15 months duration. Commence on 01/05/2018 to 31/07/2019.

NIHR Newcastle BRC reference number: BH Ref 173606 / PDB053

*Main contact*: Wendy Mitson, Financial Accountant

Newcastle upon Tyne Hospitals

Accounts Payable Department, Research Finance

Regent Point

Regent Farm Road

Gosforth

Newcastle, NE3 3HD

**IRAS project ID**: 241266

**Ethical Approval**

North East – Newcastle & North Tyneside 1 Research Ethics Committee (REC reference: 18/NE/0265 – favourable REC opinion gained on 25/09/2018)

**Protocol approval**

MIlk Intervention Muscle AgeiNg (MIlkMAN): pilot study

BH Ref 173606 / PDB053

**Signatures**

By signing this document, I am confirming that I have read, understood and approved this protocol for the above study.


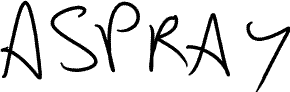
 02/01/2019

Dr Terry Aspray _________________________________________ ______________________

Chief Investigator Signature Date


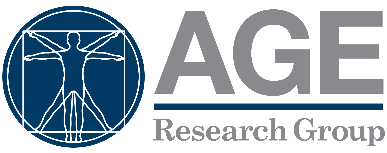


Contents

[Chief Investigator 1](#_Toc513124680)

[Principal Investigator (main study contact) 1](#_Toc513124681)

[Co-investigators 1](#_Toc513124682)

[Sponsor 2](#_Toc513124683)

[Funder 2](#_Toc513124684)

[Synopsis 4](#_Toc513124685)

[Background and rationale 6](#_Toc513124686)

[Study aims 8](#_Toc513124687)

[Primary aims 8](#_Toc513124688)

[Primary and secondary outcomes of the study 8](#_Toc513124689)

[Study duration 9](#_Toc513124690)

[Inclusion/ exclusion criteria 9](#_Toc513124691)

[Recruitment 10](#_Toc513124692)

Randomisation & [Consent 11](#_Toc513124693)

[Flow diagram of the study 1](#_Toc513124694)2

[Data collection](#_Toc513124695) 13

[Ethical considerations and risk and burden 18](#_Toc513124696)

[Adverse events (defining, recording, reporting)](#_Toc513124697) 19

[Data analysis and statistical methods 20](#_Toc513124698)

[Dissemination](#_Toc513124699) 21

[Key milestones 21](#_Toc513124700)

[References](#_Toc513124701) 21

[Useful readings 24](#_Toc513124702)

[Appendices](#_Toc513124561) 25

**Synopsis**

Sarcopenia is a progressive decline in skeletal muscle mass, strength and function with ageing, which puts older adults at increased risk of falls, frailty, disability, loss of independence and death. Sarcopenia is a major clinical problem that leads to increased use of health and social care. The loss of muscle mass and strength with ageing can be accelerated by other risk factors such as disease, physical inactivity, reduced mobility, and poor diet.

Adequate intake of dietary protein (amino acids, AA) in combination with resistance exercise (RE) are recognised as key modifiable factors in muscle ageing and physical decline. Intervention studies that investigated a combined effect of protein supplements (AA) and RE on muscle have observed that greater amounts of protein (~20g per meal) consumed repeatedly after exercise may result in increase in muscle mass in young and older adults. However, current research lacks in understanding the role of whole foods (e.g. milk and dairy products, fish, and meats) in attenuating the loss of muscle mass and function in older adults. Milk (cow) is a source of high quality protein (whey and caseins), minerals (calcium, phosphorus, magnesium), vitamins (A, D, E, B complex), and bioactive fats, and has many health benefits (e.g. reduced risk of diabetes, cardiovascular diseases, and obesity). Ratio of these nutrients, especially proteins and milk fat may be important for muscle health. Specifically, consumption of whole milk has been linked to reduced muscle soreness and damage after exercise, and better absorption of AA in the presence of milk fats and greater production of muscle proteins in young adults and athletes. Therefore, regular intake of high quality, nutrient-dense food such as whole milk in combination with RE may provide a platform for developing a strategy for maintenance of healthy muscles in older adults that does not involve drugs or medical products.

This pilot study will recruit 30 older adults aged 65 and over (≥65) who live in the community We will include individuals who have no known milk allergies and do not currently participate in structured gym program, and meet other inclusion and exclusion criteria as reviewed through their registering general practice within North East and North Cumbria Clinical Research Network, North East England. Eligible individuals will be randomly assigned to one of 3 groups: (group 1) ‘whole milk + RE’, (group 2) ‘skimmed milk + RE’, and (group 3) ‘control drink + RE’ (juice with added carbohydrates to adjust for energy in whole milk). Each 500ml of milk provides about 20g of protein. Participants in each group will receive a home visit from a trained researcher to complete sociodemographic and health questionnaire and functioning assessment over a period of 6 weeks (approximately). Next, they will complete a 6-week intervention (milk/ control drink + RE) twice a week at a sports/recreation centre under the supervision of an exercise physiologist. Right after exercise, participant will consume 500ml milk/ control drink within 40-45 minutes (supervised), and another 500ml at home over the next 4-5 hours (unsupervised). Another home visit will be conducted after the intervention is completed over a 3-4 week period (approximately), and will include similar health and functioning assessments from the first home visit, and will also gather participant views about participating in the study.

Study aims:

(i) Feasibility and acceptability of (whole) milk in combination with resistance exercise as an intervention to increase muscle function (physical performance), strength and mass in community-dwelling older adults aged ≥65 who may be at risk of sarcopenia. We will ask:

- Is an intervention of 2 × 500ml milk + RE twice a week for 6 weeks feasible?
- Is an intervention of 2 × 500ml milk + RE twice a week for 6 weeks acceptable to community-dwelling older adults?

(ii) To explore whether consumption of milk (whole and skimmed) in combination with RE has an influence on physical performance, muscle strength, muscle mass, self-reported quality of life, and activities of daily living in community-dwelling older adults who may be at risk of sarcopenia compared to control group (juice supplemented with carbohydrates + RE)?

(iii) To provide important information for planned future research.

# Background and rationale

#

The UK population is rapidly ageing; the number of adults aged ≥65 increased by 17.3% in the last decade, and in 2016 was estimated to account for 18% of the total population of 65.6 million^1^. The extraordinary gain in human longevity and the fast growth of the older population worldwide have been regarded as among the greatest accomplishments of humanity, but they are also a cause for concern and a societal challenge. The main challenge will be to maximise the potential for these extra years to be ‘healthy years’, and minimise burden from disease, disability, and dependency^2^. Genetic and non-genetic factors, such as smoking, physical activity and diet contribute to the heterogeneity in the ageing experience. There is a substantial evidence from experimental and observational studies to support the role of specific foods, dietary patterns, and nutrients in the prevention of chronic diseases and mortality^3^, and in improving the quality of life with ageing^4^. Understanding the important influences and mechanisms of healthy ageing such as diet and physical activity for optimising health and wellbeing in later life is essential for the development of effective interventions.

There is an increasing interest in understanding influences of sarcopenia, defined by both the European Working Group on Sarcopenia in Older People (EWGSOP) and a United States International Working Group as a progressive loss of skeletal muscle mass, strength and function with ageing^5^. Sarcopenia is strongly associated with adverse health outcomes in older adults, including frailty, falls, hospitalisation, disability and death^6^. The onset of sarcopenia is a central confounder for health of an older person, because skeletal muscle accounts for 40% total body mass, and serves as a vital protein store and metabolic regulator, in addition to its primary function related to posture, breathing, and mobility^7^. The prevalence of sarcopenia increases with advancing age, and reaches over 20% in very old adults (aged 85 and over)^8^. Costs associated with sarcopenia such as falls to the NHS are estimated to be more than £2.3 billion a year (NICE, 2013)^9^, emphasising the need for sustainable preventive measures aimed to preserve muscle health and function in a rapidly ageing population.

The loss of muscle mass and strength associated with ageing is further accelerated by acute and chronic stressors such as disease, physical inactivity, reduced mobility, and poor diet. Adequate intake of dietary protein (essential amino acids; EAA) from animal and plant sources in combination with resistance exercise (RE) are recognised as key modifiable factors in muscle ageing and physical decline^10,11^. Intervention studies that examined a combined effect of protein (EAA) supplements and RE to stimulate muscle protein synthesis (MPS) have observed an increase in total muscle protein by protein intake within 3-5 hours after exercise in both young and older adults^12^. Older adults experience a blunted response after protein ingestion to stimulate MPS, especially to lower amounts of protein (EAA) of <20g (<10g) compared with young adults. It has been shown that greater amounts of protein supplementation and periodic feeding in combination with repeated bouts of RE may results in increased muscle mass in older adults diagnosed with frailty and sarcopenia^13-15^.

However, current research lacks in understanding the role of whole foods (e.g. milk and dairy products, fish, and meats) in the prevention of sarcopenia in older adults, although the importance of a ‘whole food approach’ and the combined effects of nutrients within foods on health and functioning is well recognised^16^. Regular consumption of high quality, nutrient-dense foods, rich in macro- and micronutrients relevant for muscle health^17^ within a varied diet may provide a platform for developing strategies for maintenance of muscle strength, mass and function in older adults that do not include drugs, supplements and medical products.

Milk (cow) is an example of whole foods which may have potential to slow down and prevent muscle wasting. Whole milk is a source of high-quality proteins (6.3g/l (20%) of soluble whey proteins and 26g/l (80%) of insoluble caseins), minerals (e.g.1200 mg/l of calcium, 950 mg/l of phosphorus, 120 mg/l of magnesium), vitamins (fat-soluble A, D, and E and water-soluble B vitamins and vitamin C), bioactive lipids and fatty acids of which 30% are (mono and poly) unsaturated and 70% saturated fatty acids^18^. Whey protein supplements are considered superior to other protein sources to stimulate MPS after exercise in young and older adults^19^. Furthermore, intake of milk fats in whole milk increased the use of EAA for muscle protein synthesis after exercise in young men compared to fat-free milk^20^, suggesting additional benefits of milk lipids for muscle. Other benefits of milk (with milk fat) for muscle health include the reduction in exercise-related muscle damage, soreness, and decline in muscle performance in young adults^21^. Therefore, in addition to proteins, whole milk contains valuable minerals, vitamins, carbohydrates and milk fats (a delivery medium for fat-soluble vitamins A, D and E)^18^, and their ratio may be important for ageing muscle. Studies comparing the impact of low-fat versus whole milk providing >20g protein/day after exercise on muscle mass and function in older adults are lacking.

Milk and dairy product have other health benefits as an important part of a healthy diet across the life course, being crucial not only for the maintenance of bone density, but also associated with a reduced risk of adverse health outcomes, including diabetes, cardiovascular diseases, dementia, micronutrient deficiency, and obesity.

Taken together, whole milk (a functional food) in combination with RE could be beneficial for muscle health and function in older adults at risk of sarcopenia, and an important part of sarcopenia prevention in later life. Therefore, the main aim of this study is to explore the feasibility and acceptability of such an intervention in relation to physical performance, muscle strength and mass in community-dwelling older adults who may be at risk of sarcopenia.

This study is founded by the NIHR Newcastle Biomedical Research Centre, Newcastle University and Arla®, and sponsored by the Northumbria Healthcare NHS Foundation Trust, North Tyneside General Hospital (NTGH). The research team of the study has a combined expertise in geriatric^22,23^ and sports medicine^21^, gerontology^24,25^, nutritional interventions^21^ and population-based studies with older adults^24,25^. The host institutions (NIHR Newcastle Biomedical Research Centre and Institute of Neuroscience, Newcastle University) are recognised for their interdisciplinary research in ageing through translational pathway, and excellent collaborations on national and international scale.

**Hypotheses**

1. Consumption of whole milk (2 × 500ml; 2 × ~20g protein) after a RE session twice a week over the period of 6 weeks post-randomisation is feasible as an intervention in older adults (aged ≥65) who may be at risk of sarcopenia.
2. The intervention is acceptable with good compliance, low attrition, and no adverse health effects, including appetite suppression, negative dietary changes, muscle damage and soreness.

# Study aims

# Primary aims

1. Examine the feasibility and acceptability of (whole) milk in combination with RE as an intervention to increase muscle function (physical performance), strength and mass in community-dwelling older adults (aged ≥65) who may be at risk of sarcopenia through the following research questions:
2. Is an intervention of 2 × 500ml milk + RE twice a week for 6 weeks feasible?
3. Is an intervention of 2 × 500ml milk + RE twice a week for 6 weeks acceptable to community-dwelling older adults?
4. Provide essential data for planned future substantive research.

Secondary aim

1. Explore whether consumption of milk (whole and skimmed) in combination with RE has an influence on physical performance, muscle strength, muscle mass, and self-reported quality of life in community-dwelling older adults (aged ≥65) who may be at risk of sarcopenia.

# Primary and secondary outcomes of the study

Primary outcome measures

1. Feasibility and acceptability of the intervention (Time frame: 6 weeks post-randomisation and baseline assessment).
2. Measures to include: applicability, dosage and duration of the intervention, compliance, attrition, and adverse health effects such as appetite suppression, dietary changes and muscle soreness (Time frame: 6 weeks post-intervention).

Secondary outcome measures for exploratory analyses

1. Difference in physical performance measures: Short Physical Performance Battery^26^ (balance, 4m-gait speed, 5-chair stands) (Time frame: at baseline and after 6 weeks of intervention).
2. Difference in muscle strength measure: maximum strength test^27^ (grip strength, GS; Jamar hand-held 5030J1 dynamometer) (Time frame: at baseline and after 6 weeks of intervention).
3. Difference in muscle mass measure: Bioelectric Impedance Analysis (BIA; Tanita MC-780MA Body Composition Analyzer) (Time frame: at baseline and after 6 weeks of intervention)^28^.
4. Difference in self-reported quality of life measure: SF-12 (Time frame: at baseline and after 6 weeks of intervention)^29^.
5. Difference in activities of daily living measure: Barthel Index^30^.

# Study duration

The study will be conducted over 14 (maximum 15) months in total (01/05/2018 –19/08/2019). The end of the ‘active’ study period will be marked by randomisation of 30 eligible participants into the three intervention groups (i.e. (group 1) ‘whole milk + RE’; (group 2) ‘skimmed milk + RE’, and (group 3) ‘control drink’ + RE) and data collection from: (i) health and functioning assessments (baseline and post-intervention); (ii) nutrition + exercise intervention, and (iii) participants’ feedback about the study.

Although the end of the study is currently marked by the end date of funding (19/08/2019), we anticipate that this pilot study will lead to a main study funding application—as given within the main aims of the study.

# Inclusion criteria

1. Older adults aged ≥65 living in the community (not in care homes)
2. Registered patient with one of the General Practitioner (GP) surgeries within the NIHR North East and North Cumbria Clinical Research Network (CRN).

**Exclusion criteria**

1. Lacks capacity to consent—capacity is essential given the nature and demands of the research and the aim to gain participant feedback of the research experience
2. Self-reported lactose intolerance or milk allergies
3. Dislikes milk
4. Unable to understand instructions for muscle strength and function assessments in English or unwilling to participate in protocol when explained
5. Dislikes gym exercise with equipment
6. Doctor diagnosed diabetes I and II
7. Doctor diagnosed impaired renal function (estimated glomerular filtration rate <30ml/min/1.73m^2^)
8. Doctor diagnosed liver function impairment (e.g. viral hepatitis, alcoholic hepatitis, liver cirrhosis, biliary obstruction, non-alcoholic fatty liver disease, ischemic liver injury)
9. Significant respiratory disease (e.g. COPD, severe asthma, and bronchitis)
10. Doctor diagnosed gastrointestinal tract disease (e.g. gastritis, peptic ulceration, inflammatory bowel disease, gastric carcinoma, pancreas disease)
11. History of neuromuscular problems and all other co-morbidities that substantially interact with mobility and muscle metabolism (e.g. severe arthritis, rigidity, paralysis)
12. Pacemaker or severe heart failure or any other significant heart disease
13. Uncontrolled hypertension (160/100) and uncontrolled hypotension (<100 systolic)
14. Hip or knee replacement
15. An individual who the research team (i.e. exercise physiologist) evaluates as not suitable for the intervention because of safety reasons
16. BMI ≥30kg/m^2^
17. Unintentional weight loss ≥5kg for the last 3 months
18. Taking warfarin (because of possible interaction with control drink (cranberry juice))
19. Structured resistance exercise training and gym-based programme in last month
20. An individual who the General Practitioner feels it is inappropriate for the researchers to approach—the general practitioner has detailed knowledge of patients and may consider individual unsuitable for approach for reasons such as end stage terminal disease or safety risk such as any medical and physical conditions that preclude safe participation in an exercise programme.

Recruitment (main team member(s) responsible: Dr Antoneta Granic)

Using inclusion/ exclusion criteria potential participants will be identified through multiple general practices in North East and North Cumbria CRN. Five recruitment sites were pre-screened for feasibility by North East and North Cumbria CRN (through local CRN support), and two GP practices in North Tyneside Clinical Commissioning Group (CCG) provided feedback during the application for the study funding.

Recruitment will be organised in two stages: pre-screening and screening. The pre-screening stage will first identify potential participants from participating general practices (GPs), who have agreed to review their patient database using predetermined exclusion/ inclusion criteria provided by the study team. For those GP patients who meet the criteria, surgeries will post recruitment packs on the behalf of the research team. Recruitment packs include: an invitation letter from the GPs, a Participant Information Sheet providing information about the study, a cover letter from the research group, a reply slip where individuals can indicate if they are interested in hearing more about the study, and a pre paid return envelope. The pre-screening stage then invloves a researcher telephoning those individuals who have expressed an interest in hearing more about the study via the reply slip and answering any questions they may have. With their permission the researcher will also conducting a 5-item SARC-F questionnaire^31^  to assess safety to proceed in the study and evaluate the use of SARC-F as a pre-screening tool for the inclusion of older adults in community-based studies involving resistance exercise program. The SARC-F questionnaire has five components (0-2 points for each) that assesses any difficulties with the following: (i) strength; (ii) assistance with walking; (iii) rise from a chair; (iv) climbing stairs, and (v) falls in the last year (Appendix 1).

Individual assessed to be unsafe to proceed will be informed in detail why they are not eligible for the study, thanked for their time and asked about the possibility to take part in other studies conducted by the AGE Research Group led by Professor Avan A Sayer.

Individuals assessed to be safe to proceed will be offered a home-based screening assessment, where possible this will be arranged at a day and time to suit potential particiapnts and their families if requested. This visit will be used to evaluate inclusion/ exclusion criteria not screened through general practices. The researcher will also measure participants’ grip strength and walking speed to evaluate their muscle strength and function based on the establish cut-offs (low grip strength: <20 kg in women, and <30 kg in men^5^; low walking speed: <0.8 m/s or ≥5 s over 4 m distance^5^). This information will serve two purposes: (i) for comparison with SARC-F results, and (ii) to allow equal distribution of those with some muscle strength weakness across intervention groups. For grip strength assessment, each participant will perform six trials (three in their right and three in their left hand, alternating between the hands), and the final grip strength value will be expressed as the maximum of the six trials. Each participant will complete walking speed assessment once.

The study aims to recruit 30 participants (15 men and 15 women) aged ≥65 years who meet the pre-screen and screening criteria. A reserve list of 6-9 eligible individuals will also be created to factor in replacement of those who withdraw early from the study.

**Randomisation** (main team member(s) responsible: Dr Antoneta Granic)

Thirty participants will be allocated to one of the three intervention groups using a minimisation algorithm by an independent researcher. This will ensure the equal allocation of participants between the groups, so that gender and muscle strength (grip strength assessed at the screening assessment) are equally distributed and balanced between the groups. The researcher will use a free, open-source minimisation software (e.g. MiniPy 0.3, http://minimpy.sourceforge.net or OxMaR, www.ccmp.ox.ac.uk/oxmar), which features elements of randomness in the minimisation algorithm^32,33^. Briefly, the first subjects are allocated randomly into one of the interventions. Allocation of the subsequent participants will be based on hypothetical stepwise allocation of each individual to every group and computation of the imbalance score corresponding to each allocation. The imbalance scores will be compared and participants will be allocated to the group corresponding to the least imbalance score (preferred group). To ensure an element of randomisation, the participant is usually allocated to the preferred group with a higher probability (denoted as P_h_), and to other groups (non-preferred group) with lower probabilities (denoted as P_l_)^32,33^.

Because of the nature of the intervention, it is not possible to blind participants to intervention groups. The researcher team will not be blinded to nutritional part of the intervention.

Consent (main team member(s) responsible: Dr Antoneta Granic)

Initial consent will be obtained by a researcher visiting the participants within their own home and conducting the screening assessment prior to randomisation to the study. The researcher will be suitably trained to conduct a 5-step informed consent process. The participants will be given enough time as needed to consent. The researcher must inform the participant that they are free to reconsider and withdraw from the study at any time. The notion of process consent will be implemented throughout the participants active research phase (i.e. from baseline to post-intervention assessment). Valid enduring consent will be assessed at the baseline assessment, intervention and post-intervention home visits. If a participant loses capacity to consent during the research process, he/she will be withdrawn from the study.

# Flow diagram of the study

A flow diagram of the study protocol with timelines is outlined in *Figure 1*. We anticipate that the recruitment (assessment of eligibility) up to allocation (randomisation) will be finalised within 2-3 months. The feedback from two general practices data base searches revealed, on average, 430 eligible individuals per practice based on predetermined exclusion/ inclusion criteria (excluding criteria that will be evaluated by the research team over the telephone and during the home-based screening assessment) ) (local CRN support, personal communication). The aim is to start the intervention with a (pre)-screened, eligible group of 30 participants randomised into 3 groups (‘whole milk + RE’; ‘skimmed milk + RE’; ‘control drink + RE’), and immediately assessed for baseline health and functioning (i.e. home-based baseline assessment) within 6 weeks (approximately), followed by 6 weeks of intervention. Post-intervention assessments at participants’ home will be conducted the following week after the intervention completion, and will be finalised within 3 to 4 weeks. Participants’ feedback will be collected at the end of post-intervention assessments. Analysis of data reporting of the results will be completed within 2-3 months after the study active duration of data collection (from randomisation to post-intervention assessment).

Intervention is fixed to 6 weeks for each participant, whereas recruitment, baseline and post-intervention assessments (including participant feedback) may be conducted prior to estimate times. Specifically, the recruitment (assessment of eligibility) up to allocation (randomisation) may be completed within 2 months.


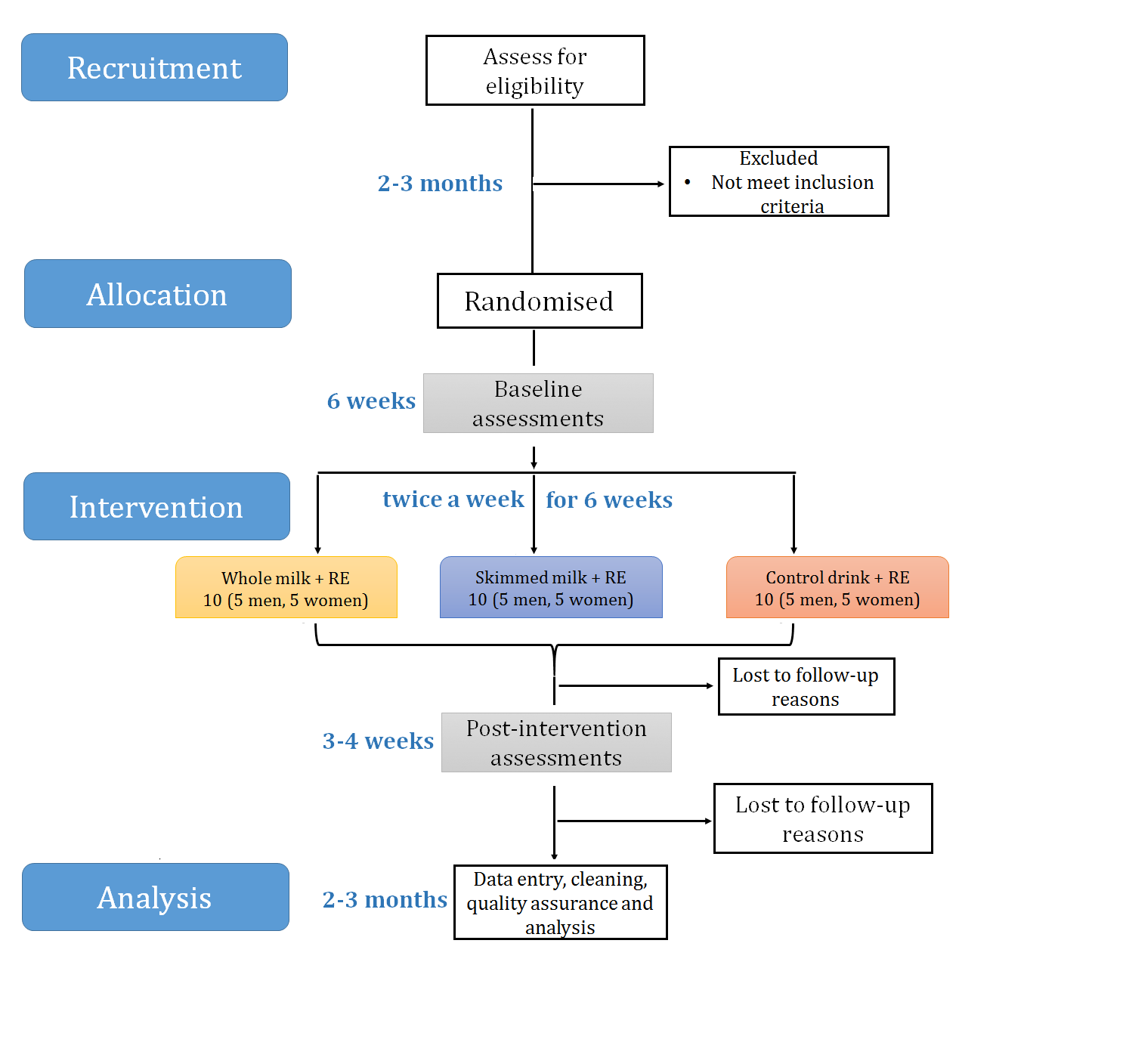


*Figure 1*. Flow diagram of the study from enrolment to data analysis.

The active duration for data collection for each participant will span approximately 10 weeks. An example of participant’s journey through the study after randomisation is presented in *Figure 2*.

1. Week 1: verifying consent and home-based baseline assessment
2. Week 2 or 3 until Week 7 or 8: verifying consent and 6-week intervention
3. Week 9 or 10: verifying consent and post-intervention home-based assessment.

Except for intervention (6 consecutive weeks), this time scale can be adjusted to participants’ individual needs with maximum 2-3 weeks gap between baseline assessment and the first week of intervention, and maximum 2-3 weeks gap between the last week of intervention and post-intervention assessment.


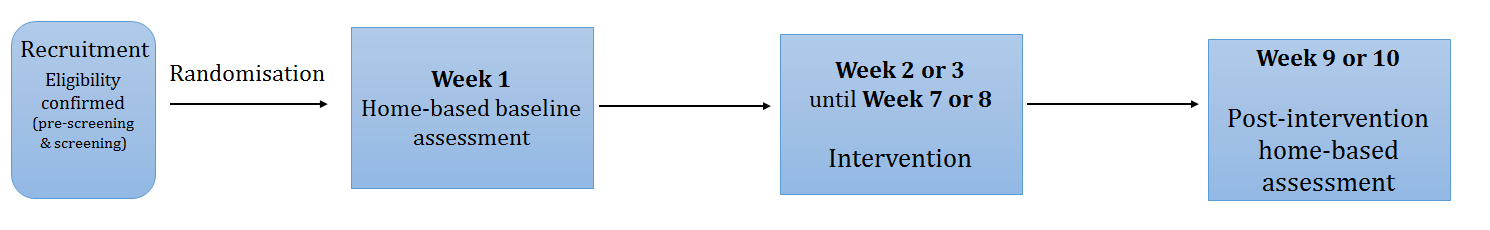


*Figure 2*. Flow diagram of participants’ journey through the study.

**Data collection**

- HOME-BASED SCREENING ASSESSMENT (main team member(s) responsible: Dr Antoneta Granic)

Researchers trained in assessment with older adults will visit potential participants selected through general practices and pre-screened with the SARC-F (i.e. evaluated by the research team as safe to proceed) in their own home to carry out screening for other exclusion/ inclusion criteria not assessed at pre-screening (e.g. milk allergies, participation in structured resistance exercise in the last month). Grip strength and walking speed will be measured to establish participants’ muscle strength and function status based on the established cut-offs^5^. An informed consent will be conducted before the assessments. Eligible individuals will be informed about the study procedure and their journey through the study (from randomisation to post-intervention assessment). Non-eligible individuals will be explained why they did not qualify for the study, thanked for their time, and will be informed about the possibility to participate in other studies led by Prof Avan A Sayer (AGE Research Group).

This visit with the informed consent will last about 35 (maximum 40) minutes.

- HOME-BASED BASELINE ASSESSMENT: HEALTH AND FUNCTIONING PROFILE (main team member(s) responsible: Dr Antoneta Granic)

Trained researchers will visit 30 participants enrolled in the study in their own home to conduct in depth interview and baseline assessments of the following domains:

1. Sociodemographic profile: age; sex; marital status; education; social class, and multiple index of deprivation
2. General health and functioning: SF-12 Health Survey^29^; self-reported diseases diagnosed by a doctor; list of medication (prescribed and over-the-counter); Mini Mental State Examination; Geriatric Depression Scale (15-item version) and Barthel Index^30^ (Activities of Daily Living); blood pressure (systolic and diastolic)
3. Dietary intake: 24-hr dietary recall (Intake24 (https://intake24.co.uk/) multi-pass, computerised 24-hr recall supported by the Newcastle University)
4. Appetite assessment: a 4-item Simplified Nutritional Appetite Questionnaire^34^
5. Self-reported physical activity: questionnaire assessing the frequency and duration of low intensity, moderate intensity and vigorous intensity physical activity, as assessed in the Newcastle 85+ study^35^
6. Anthropometry: demi-span (sex-specific height calculation), waist, hip and calf circumference
7. Muscle strength: maximum grip strength measured 3 times in each arm
8. Physical performance: Short Physical Performance Battery^26^ (balance, 4m-gait speed, 5-chair stands)
9. Muscle mass (body composition), as measured by Bioelectric Impedance Analysis (BIA; Tanita MC-780MA Body Composition Analyzer).

Baseline assessments of blood pressure and muscle mass (body composition) will be conducted at the intervention site (The Parks, Contours gym) before the first RE session, and dietary intake and appetite will be assessed during the post-exercise recovery period. Body analyser will be parts tested by the NTGH.

Detailed health and functioning profile will involve minimal risk and inconvenience to participants and will be conducted under 70 minutes (including verification of the informed consent and excluding assessments done at The Parks).

- INTERVENTION: MILK/ CONTROL DRINK + RESISTANCE EXERCISE (main team member(s) responsible: certified exercised physiologist and Prof Emma Stevenson)

Thirty participants will be randomized into three groups (10 each; 5 men and 5 women):

1. ‘whole milk + RE’;
2. ‘skimmed milk + RE’, and
3. ‘control drink’ (juice supplemented with carbohydrates) + RE’.

Resistance exercise programme

RE will be conducted over 6 weeks (twice a week on two non-consecutive days) at The Parks, Contours gym & fitness suite, North Shields, North Tyneside Council in a small group of 2-3 participants, and will be supervised by a trained and experienced exercise physiologist. Participants will be advised to resume their usual diet, and to have their regular breakfast before the intervention. Four sets of 8-12 repetitions (upper and lower body) exercises at the sub-maximal level of effort (70-79% 1-repetition maximum) will be performed after a short warm-up exercise (e.g. 5 minutes of stretching exercises, walking or stationary cycling)^36,37^. Each set of RE will be followed by 1-2 minute of resting period, and a rest of 4 seconds between repetitions^36^. Each participant will receive a booklet with diagrams and short instructions of exercise programme (RE booklet). To increase their engagement in exercise, each participant will have the opportunity to record in the RE booklet how many repetitions and how much weight for each set of exercise they have completed and lifted.

Exercise intensity, volume, frequency and duration have been determined based on the recent literature^36-38^, and the American College of Sports Medicine (ACSM) recommendations for older adults^37^. Details about the RE programme over 6 weeks are outlined in the RE protocol (MIlkMAN_Resistance Exercise Protocol_v.1-20-07-18). The complete RE session will be finalised within 1 hour, except the first RE session, which will last 1 hour and 30 to 40 minutes and include a short induction programme at the gym detailed below. Blood pressure and heart rate will be measured pre and post each RE session. Blood pressure classification and cut-offs for the blood pressure change pre and post-RE will be based on the guidelines provided by the American College of Cardiology/American Heart Association Task Force^39^ and on the published literature^40-42^. For older adults aged 65 and over blood pressure of 130/80 mmHg is considered as optimal^39^. Exercise physiologist will record changes in blood pressure and heart rate pre and post each RE session. If participant’s blood pressure approaches the exclusion criteria for the study (>160/100 mmHg for hypertension and <100mmHg systolic for hypotension) before exercise, the Chief Investigator (a consultant physician) will be notified and consulted. Changes that are regarded to be adverse events will be recorded and reported to the Chief Investigator (Dr Terry Aspray) for evaluation and further adverse events reporting (described below). Any other health risks will be closely monitored by exercise physiologist. Post-exercise differential ratings of perceived exertion (RPE) ~ 10 minutes after the completion of exercise will be assessed using the CR100® scale^43^ (Appendix 2). The scale offers finely graded numerical range of 0-100 arbitrary units (AU) with several verbal anchors next to the scale corresponding to whole numbers (0 ‘nothing at all’; 12 ‘easy’; 22 ‘moderate’; 35 ‘somewhat hard’; 50 ‘hard’; 70 ‘very hard’; 100 ‘maximal’). Participants will be asked to self-asses their level of perceived exertion for the overall session, and upper-body and lower-body muscles. Muscle soreness will be assessed with a simple visual analogue scale (Appendix 3) ~40-45 minutes after each RE session. Additionally, each participant will be called at home in the evening (up to 6-7 hours post-RE) to evaluate his/ her muscle soreness using the same visual analogue scale.

During the intervention, participants will be advised to resume their regular level of physical activity and lifestyle habits and not to participate in any other activities provided with the gym membership during the intervention.

Facility

The supervised RE programme will be conducted in the community at a sports/ recreation centre (e.g. The Parks, Contours gym & fitness suite, North Shields, North Tyneside Council). The RE equipment for upper and lower body exercises will be provided by the centre, and will be used as a part of free monthly membership (£60 for 6 weeks/participant paid by the study grant). Each participant will complete a short medical questionnaire provided by the gym, sign the Contours gym membership terms and conditions agreement (<https://my.northtyneside.gov.uk/sites/default/files/web-page-related-files/CONTOURS%20MEMBERSHIP%20TERMS%20AND%20CONDITIONS%202016.pdf>), and undergo a short induction programme

(<https://my.northtyneside.gov.uk/sites/default/files/web-page-related-files/Contours%20Gym%20Inductions.pdf>) before the commencement of the first intervention session. The Contours instructor will conduct only the first part of the induction programme and explain the use of gym facilities and resistance machines to each participant (contact person: Richard Lazonby, GP Referral Instructor, Environment, Housing, Leisure, North Tyneside Council; [Richard.Lazonby@northtyneside.gov.uk](mailto:Richard.Lazonby@northtyneside.gov.uk) ). A private room with a fridge (parts tested) will be provided at the Parks for the participants’ recovery and milk drinking (500ml) during the duration of the intervention.

Milk and control drink intake

Milk / control drink will be consumed as a bolus intake of 500ml under the supervision of a researcher immediately after exercise over 40-50 minutes during the recovery period (or longer if needed). Participants will be advised to resume their usual diet. Second dose of 500ml will be consumed at participants’ home over the next 4-5 hours. All drinks consumed at home will be clearly labelled as either ‘MIlkMAN study milk’ or ‘MIlkMAN study juice’. Second dose of 500ml can be consumed with other foods as a part of their usual diet (e.g. tea, cereal, mushed potatoes, etc.). Participants will be contacted by phone in the evening to check for muscle soreness and compliance with milk/ control drink consumption. Each participant will be provided with a plastic measuring jug (500ml) and a log sheet to measure and record milk/control drink consumption at home and to report it back to a researcher conducting the phone call.

On average, 500ml milk contains ~20g of protein needed to stimulate muscle protein synthesis above stimulation provided by RE. Whole (cow) milk (nutritional estimates of 22 UK samples during winter and summer) provides 66 kcal/100g of energy^44^. Fresh milk will be provided by Arla® (Nutrition Specialist: Lise Larsen at Arla Foods). Arla Cravendale® whole milk contains 3.6g fat, 3.4g protein, and 4.7g of carbohydrates per 100g of milk. Arla Cravendale® skimmed milk contains 0.3g fat, 3.6g protein, and 4.9g of carbohydrate per 100g of milk. Energy of control drink (cranberry juice; Ocean Spray Classic) will be balanced to match whole milk energy content and supplemented with maltodextrin (e.g. cranberry juice (45kcal/100g of energy^44^) will be supplemented with 6.05g of maltodextrin (3.8kcal/g) per 100g of juice; 1tsp = 15kcal; 30.25g for 500ml of juice).

Milk and control drink storage and handling

Milk (whole and skimmed) will be provided by Arla® (contact person: Lise Larsen, Senior Nutrition Specialist, Arla Foods, Aarhus, Denmark; lise.larsen@arlafoods.com) in packs of either 500ml or 1 litre on a bi-weekly basis through a local distributor (Tesco or Sainsbury’s), and refrigerated at the Campus for Ageing and Vitality, Newcastle University for a long-term storage (parts tested fridge with a lock). Arla Cravendale® milk stays fresh for 21 days if left unopened. Control drink (cranberry juice) will be bought fresh weekly (Tesco) and refrigerated in a similar fashion. Control drink will be supplemented with maltodextrin at the day of intervention by a research team member. During the intervention period of the study, a researcher not involved in exercise intervention will transport milk/ control drink in cooler boxes to The Parks on daily basis. At The Parks, a fridge (parts tested) will be provided in a designated recovery room for a short-term storage of milk/ control drink.

The total time needed to complete the first visit at The Parks is about 2 and half hours (gym induction programme, assessments, intervention and recovery). This first visit will include blood pressure, body composition, diet and appetite assessment as a part of baseline assessment. The total time needed to complete the remaining RE + drink intake sessions (including recovery period) is 1 hour and 45 minutes. For participants who need more rest, additional times will be provided. The last (12th) visit at The Parks will include body composition, blood pressure, diet and appetite assessment after exercise as a part of post-intervention assessment and will last 2 hours and 10 minutes.

Individual transport will be provided to transport participants to The Parks and back home.

Those who prefer to use public or personal transport or to walk will be reimbursed for their fare.

- HOME-BASED POST-INTERVENTION ASSESSMENT (main team member(s) responsible: Dr Antoneta Granic)

A post-intervention visit, in the participants own home, will be arranged after 6 weeks of intervention to assess the following domains:

1. Physical performance: Short Physical Performance Battery (balance, 4m-gait speed, 5-chair stands)
2. Muscle strength: maximum strength test (grip strength, GS; Jamar hand-held 5030J1 dynamometer)
3. Muscle mass: Bioelectric Impedance Analysis (BIA; Tanita MC-780MA Body Composition Analyzer)
4. Dietary intake: 24-hr dietary recall (Intake24 (https://intake24.co.uk/) multi-pass, computerised 24-hr recall supported by the Newcastle University)
5. Appetite assessment: a 4-item Simplified Nutritional Appetite Questionnaire
6. General health and functioning: Self-reported quality of life: SF-12; Activities of daily living: Barthel Index; blood pressure (systolic and diastolic)

Participants’ feedback will be collected at the end of follow-up assessment using a combination of structured multiple-response and standardised open-ended questions to explore the following themes:

1. Attitudes and barriers of 2 × 500ml milk intake post-exercise (e.g. volume of liquid, taste, etc.)
2. Their opinion about milk as a functional food for muscle strength/ function
3. Appetite changes, changes in habitual diet because of milk/ control drink intake
4. What they liked and disliked about the study (intervention).

The follow-up visit will be completed within 50 minutes.

Muscle mass (body composition), appetite assessment (a 4-item Simplified Nutritional Appetite Questionnaire), dietary intake (24-hr dietary recall) and blood pressure will be assessed at the last (12^th^) intervention visit during the 45 minutes recovery period at The Parks.

# Ethical considerations and risk and burden

As with all research involving human subjects the wellbeing and safeguarding of individuals is paramount.

All researchers who have direct contact with participants will have all of the necessary approvals in place to include research passport, health checks, employment contracts, disclosure and barring service checks and will be fully trained to undertake the examinations included within the study.

Interview and other data (e.g. bioelectrical impedance, dietary intake) will be pseudonymised using a unique identifier at the time of collection. For long term storage and analysis, pseudonymised data will be encrypted and compressed, and retrievable only by members of the core research team. All anonymised data will be securely stored within a fire-walled password protected domain. Only core team member responsible for data analysis can link pseudonymised data back to individual participants.

As stated above, this study will exclude individuals who do not have capacity to consent. Should an individual lose consent during the ‘active’ period of this study then the participant will be withdrawn at that point. Permission to retain data gathered before loss of capacity is included in the participant consent form.

It is very unlikely that participants will experience harm by taking part in this study. There is small risk of increased muscle soreness and discomfort after each exercise session that will be carefully monitored and recorded by a trained exercise physiologist. Participants will be assessed regularly for muscle soreness and any signs of excess discomfort and stress using visual analogues scale, and will be called at home after each intervention session in the evening (i.e. twice a week over 6 weeks of intervention). Muscle injury with the proposed RE programme is highly unlikely because each participant will exercise at the sub-maximal level of effort, and will be supervised by a trained and certified exercise physiologist. The Parks (Contours gym) has health and safety procedures and indemnity insurance covered through the gym membership in the case of adverse events related to the equipment and facility use (contact person: Tony Morrissey, Operational Manager, Indoor Sport and Leisure, North Tyneside Council; [Tony.Morrissey@northtyneside.gov.uk](mailto:Tony.Morrissey@northtyneside.gov.uk) ). Any change in blood pressure and heart rate pre and post-RE will be carefully monitored and recorded.

There is also a small risk of gastrointestinal discomfort and possible unknown milk allergies (lactose or milk proteins). Participants who report any significant discomfort associated with milk or control drink intake (e.g. nausea, indigestion, diarrhoea, etc.) will be excluded from the study. In addition, there is a small risk of metabolic changes and weight gain due to the consumption of 650 kcal from whole milk and control drink (2 × week over 6 weeks) which will be counteracted by the increased energy expenditure during the RE programme. Appetite will be assessed at baseline and post-intervention, and total energy intake will be estimated using the Intake24 (computerised 24-hr dietary recall) pre- and post-intervention to examine any changes in energy intake. Also, weight and body fatness will be estimated using the Tanita Body Composition Analyzer at baseline and after the intervention.

There is also a small risk of falling during the walking test. However, the attending researcher will assess individual risk at the point of data collection (re-check exclusion criteria and endurance of consent) and omit measurements/ test if they felt it unsafe or that capacity to consent is not present or consent does not endure. It is made clear to participants that they are free to withdraw at any time. Should a decision to withdraw from the study be made, a reason for the withdrawal will be recorded. However, participants can withdraw without providing any explanation.

# Adverse events (defining, recording, reporting)

Recognising the importance of appropriately recording and reporting all Adverse Events (AE) which occur during the course of a participant’s involvement in MIlkMAN pilot study will be in accordance with National Research Committee (NRES) guidelines for non-CTIMP trials. A Decision Tree for Adverse Events Reporting for non-CTIMPs will be followed to classify ARs and their reporting (<https://www.nihr.ac.uk/our-faculty/clinical-research-staff/learning-and-development/documents/Decision%20Tree%20for%20Adverse%20Event%20Reporting%20NON%20CTIMPS.pdf>). We will use the latest Practice Guidelines Note <https://www.ntw.nhs.uk/content/uploads/2014/11/RGP-PGN-07-Safety-Reporting-V01-Iss-2-Nov-17.pdf> outlined by the Northumberland, Tyne and Wear NHS Foundation Trust policy as a template for the reporting of adverse events in non-CTIMPs.

This policy defines an AE as: any untoward medical occurrence in a study participant which does not necessarily have a causal relationship with the ‘treatment’ under study (e.g. abnormal laboratory findings, unfavourable symptoms or diseases). A Serious Adverse Event (SAE) is defined as: resulting in death, a life-threatening event, hospitalisation or prolongation of hospitalisation, a persistent or significant disability or incapacity, a congenital anomaly or birth defect, or is otherwise considered medically significant by the investigator.

All AE’s will be recorded on a case report form (CRF), the trial master file, and participants medical notes. Detailing: a description of the event, duration of AE (date(s)/times), and any actions taken.

In addition SAE’s will be reported to the study Chief Investigator (Dr Terry Aspray) and the sponsor (Northumbria Healthcare NHS Foundation Trust, North Tyneside General Hospital) by contacting the sponsor’s main contact (Peta Heslop; [Peta.Heslop@northumbria-healthcare.nhs.uk](mailto:Peta.Heslop@northumbria-healthcare.nhs.uk); R&D at the NTGH) within 24 hours of becoming aware of the event. If the Chief Investigator (Dr Terry Aspray) and senior co-investigators decide that the SAE is related to the study and unexpected then reports will be made to (i) the Newcastle Research Ethics Committee, using NHS HRA non-CTIMP safety report to REC form, within 15 days of becoming aware of the event and (ii) through the DATIX Incident Reporting System. If the event is assessed as a serious incident (SI), defined as resulting in unexpected or avoidable death, or serious harm, an R&D manager will inform the Director of Quality and Effectiveness, via the Clinical Governance and Risk Department (CGARD), as soon as possible by phone.

All SAEs involving a non-CE marked device under clinical investigation will be reported to the Medicines and Healthcare Products Regulatory Agency (MHRA) Devices Division, whether initially considered to be device related or not. SAEs involving CE-marked devices in a post-market surveillance study will be reported to the MHRA Adverse Incident Centre under the requirements of Devices Vigilance.

Any occurring SAEs will be included in the Annual Progress Report to the REC.

# Data analysis and statistical methods

The primary objectives of this pilot study are to assess the feasibility and acceptability of milk in combination with exercise as an intervention to improve muscle health in older adults aged ≥65 who may be at risk of developing sarcopenia in order to inform a main grant application. As such the sample size of this pilot study is limited to 30 participants and we do not make any claims of statistical power, especially for quantitative data. However, the data gathered will be explored for differences between the intervention and control group for the following quantitative measures after 6 weeks of intervention: physical performance, muscle strength, muscle mass, self-reported quality of life, and activities of daily living. We will evaluate the use of SARC-F tool for pre-screening of participants for whom is safe to be included in community-based intervention studies involving exercise. We will correlate data collected with SARC-F with the measures of muscle strength and function assessed at screen assessment. We will use descriptive and univariate/ multivariate statistical techniques in SPSS and Stata. Compliance with milk and control drink will be calculated as a percentage of actual consumption divided by expected consumption over the 6-week intervention. Calculations of the number of repetitions for each exercise within each RE session and the weight lifted will allow for calculation of several indices of training intensity. Participants’ experiences and views about the study will be also assessed with standardised open-ended questions. Data gathered form these questions will be analysed using qualitative analysis techniques, namely: content analysis. Content analysis involves the interpretation of the context of data whereby data is read and openly coded to break it down into emerging themes and sub-themes. A summary of the themes and sub-themes is then defined with the relevant quotations of the participant’s perspective. The research team includes extensive experience in both quantitative and qualitative data analysis.

# Dissemination

Participants and general practices

Flyer to all study participants featuring the main results of the study

Individual report to each participant (‘My muscle function and strength before and after MIlkMAN’)

Reports to general practices with abnormal results (blood pressure, BMI, fat mass, MMSE, and GDS)

Internal and external

Peer reviewed scientific journals

Internal reports

Conference presentation

Publication on publicly accessible website

Newcastle BRC Twitter

# Key milestones

1. Months 0 to 6^th^ and beyond: Develop an effective interdisciplinary collaboration, core MIlkMAN research team and industry partnership to investigate the feasibility and acceptability of the proposed intervention for sarcopenia in a population-based pilot study
2. Months 0 to 4^th^: Gain necessary regulatory approvals to take a population-based approach to recruitment, consent and randomisation of 30 study participants aged ≥65 years who may be at risk of developing sarcopenia
3. Months 5^th^ to 7^th^: Finalise screening (home-based assessment) and allocate 30 participants into three groups
4. Months 8^th^ to 11-12^th^: Determine the feasibility and acceptability of milk in combination with resistance exercise as an intervention to improve muscle health in older adults who may be at risk of developing sarcopenia
5. Months 13-15^th^ and beyond: Carry out preliminary analyses to evaluate the use of milk in combination with exercise as an intervention to improve muscle health, and to establish differences between intervention and control group in physical performance, muscle strength, muscle mass, self-reported quality of life and in activities of daily living. Check coding frames for qualitative data.
6. Month 16^th^ and beyond: Provide/disseminate essential pilot data for a substantive external project grant and future industry collaboration.

# References

1. Office for National Statistics. Population Estimates for UK, England and Wales, Scotland and Northern Ireland: mid-2016. Available online: https://www.ons.gov.uk/peoplepopulationandcommunity/populationandmigration/populationestimates/bulletins/annualmidyearpopulationestimates/latest (accessed on 10 January 2018).
2. Rowe JW, Kahn RL. Successful aging 2.0: conceptual expansions for the 21st century. J. Gerontol. B Psychol. Sci. Soc. Sci. 2015; 70: 593–596.
3. Jankovic N, Geelen A, Streppel MT, et al. Adherence to a healthy diet according to the World Health Organization guidelines and all-cause mortality in elderly adults from Europe and the United States. Am J Epidemiol. 2014; 180: 978–988.
4. Drewnowski A, Evans WJ. Nutrition, physical activity, and quality of life in older adults: summary. J Gerontol A Biol Sci Med Sci. 2001; 56: 89–94.
5. Cruz-Jentoft AJ, Baeyens JP, Bauer JM, et al.; European Working Group on Sarcopenia in Older People. Sarcopenia: European consensus on definition and diagnosis: Report of the European Working Group on Sarcopenia in Older People. Age Ageing. 2010;39(4):412-423.
6. Janssen I, Ross R. Linking age-related changes in skeletal muscle mass and composition with metabolism and disease. J Nutr Health Aging. 2005;9(6):408-419.
7. Wolfe RR. The underappreciated role of muscle in health and disease. Am J Clin Nutr. 2006;84(3):475-482.
8. Cruz-Jentoft AJ, Landi F, Schneider SM, et al. Prevalence of and interventions for sarcopenia in ageing adults: a systematic review. Report of the International Sarcopenia Initiative (EWGSOP and IWGS). Age Ageing. 2014;43(6):748-759.
9. National Institute for Health and Care Excellence (2013). Falls in older people: assessing risk and prevention. Clinical guidelines. www.nice.org.uk/guidelines/cg161. Accessed on August 11, 2017.
10. Peterson M, Sen A, Gordon P. Influence of resistance exercise on lean body mass in aging adults: a meta-analysis. Med Sci Sports Exerc. 2011;43(2):249-258.
11. Churchward-Venne TA, Holwerda AM, Phillips SM, et al. What is the optimal amount of protein to support post-exercise skeletal muscle reconditioning in the older adult? Sports Med. 2016;46(9):1205-1212.
12. Pennings B, Koopman R, Beelen M, et al. Exercising before protein intake allows for greater use of dietary protein–derived amino acids for de novo muscle protein synthesis in both young and elderly men. Am J Clin Nutr. 2011;93:322-331.
13. Witard OC, Jackman SR, Breen L, et al. Myofibrillar muscle protein synthesis rates subsequent to a meal in response to increasing doses of whey protein at rest and after resistance exercise. Am J Clin Nutr. 2014;99(1):86-95.
14. Moore DR, Churchward-Venne TA, Witard O, et al. Protein ingestion to stimulate myofibrillar protein synthesis requires greater relative protein intakes in healthy older versus younger men. J Gerontol A Biol Sci Med Sci. 2015;70(1):57-62.
15. Tieland M, Dirks ML, van der Zwaluw N, et al. Protein supplementation increases muscle mass gain during prolonged resistance-type exercise training in frail elderly people: a randomized, double-blind, placebo-controlled trial. J Am Med Dir Assoc. 2012;13(8):713-719.
16. The British Association for Parenteral and Enteral Nutrition (BAPEN). Putting Patients at the Centre of Good Nutritional Care. Available online: http://www.bapen.org.uk. Accessed on August 14, 2017.
17. Millward DJ. Nutrition and sarcopenia: evidence for an interaction. Proc Nutr Soc. 2012 Nov;71(4):566-75.
18. Pereira PC. Milk nutritional composition and its role in human health. Nutrition 2014;30:619-627.
19. Yang Y, Breen L, Burd NA, Hector AJ, Churchward-Venne TA, Josse AR, Tarnopolsky MA, Phillips SM. Resistance exercise enhances myofibrillar protein synthesis with graded intakes of whey protein in older men. Br J Nutr. 2012;108(10):1780-1788.
20. Elliot TA, Cree MG, Sanford AP, et al. Milk ingestion stimulates net muscle protein synthesis following resistance exercise. Med Sci Sports Exerc. 2006;38(4):667-674.
21. Rankin P, Stevenson EJ, Cockburn E. The effect of milk on the attenuation of exercise-induced muscle damage in males and females. European Journal of Applied Physiology 2015, 115(6), 1245-61.
22. Sayer AA, Robinson SM, Patel HP, Shavlakadze T, Cooper C, Grounds MD. New horizons in the pathogenesis, diagnosis and management of sarcopenia. Age Ageing 2013;42(2):145-150.
23. Mayne D, Stout NR, Aspray TJ. Diabetes, falls and fractures. Age and Ageing 2010, 39(5), 522-525.
24. Granic, A.; Mendonça, N.; Sayer, A.A.; Hill, T.R.; Davies, K.; Adamson, A.; Siervo, M.; Mathers, J.C.; Jagger, C. Low protein intake, muscle strength and physical performance in the very old: the Newcastle 85+ Study. Clin. Nutr. 2017, pii: S0261-5614(17)31403-6, doi: 10.1016/j.clnu.2017.11.005.
25. Davies K, Kingston A, Robinson L, Hughes J, Hunt JM, Barker SA, Edwards J, Collerton J, Jagger C, Kirkwood TB. Improving retention of very old participants in longitudinal research: experiences from the Newcastle 85+ study. PLoS One 2014;9(10):e108370.
26. Guralnik JM, Simonsick EM, Ferrucci L, et al. A short physical performance battery assessing lower extremity function: association with self-reported disability and prediction of mortality and nursing home admission. J Gerontol. 1994;49(2):M85-94.
27. Roberts HC, Denison HJ, Martin HJ, Patel HP, Syddall H, Cooper C, et al. A review of the measurement of grip strength in clinical and epidemiological studies: towards a standardised approach. Age Ageing 2011;40:423-429.
28. Moon JR, Stout JR, Smith-Ryan AE, et al. Tracking fat-free mass changes in elderly men and women using single-frequency bioimpedance and dual-energy X-ray absorptiometry: a four-compartment model comparison. Eur J Clin Nutr. 2013;67 Suppl 1:S40-46.
29. Gandek B, Ware JE, Aaronson NK, et al. Cross-validation of item selection and scoring for the SF-12 Health Survey in nine countries: results from the IQOLA Project. International Quality of Life Assessment. J Clin Epidemiol. 1998;51(11):1171-1178.
30. Wade DT, Collin C. The Barthel ADL Index: a standard measure of physical disability? Int Disabil Stud.1988;10(2):64-67.
31. Malmstrom TK, Morley JE. SARC-F: a simple questionnaire to rapidly diagnose sarcopenia. J Am Med Dir Assoc. 2013;14(8):531-532.
32. Saghaei, M, Saghaei, S. Implementation of an open-source customizable minimization program for allocation of patients to parallel groups in clinical trials. J Biomed Sci Eng. 2011;4:734-739.
33. O’Callaghan, CA. OxMaR: Open source free software for online minimization and randomization for clinical trials. PLoS ONE 2014;9(10):e110761.
34. Wilson MM, Thomas DR, Rubenstein LZ, et al. Appetite assessment: simple appetite questionnaire predicts weight loss in community-dwelling adults and nursing home residents. Am J Clin Nutr. 2005;82(5):1074-1081.
35. Collerton J, Barrass K, Bond J, et al. The Newcastle 85+ study: biological, clinical and psychological factors associated with healthy ageing: study protocol. BMC Geriatr. 2007;7:14.
36. Robinson MM, Dasari S, Konopka AR, et al. Enhanced protein translation underlies improved metabolic and physical adaptations to different exercise training modes in young and old humans. Cell Metab. 2017;25(3):581-592.
37. Peterson MD, Gordon PM. Resistance exercise for the aging adult: clinical implications and prescription guidelines. Am J Med. 2011;124(3):194-198.
38. Borde R, Hortobágyi T, Granacher U. Dose-response relationships of resistance training in healthy old adults: a systematic review and meta-analysis. Sports Med. 2015;45(12):1693-1720.
39. Whelton PK, Carey RM, Aronow WS, et al. 2017 ACC/AHA/AAPA/ABC/ACPM/AGS/APhA/ASH/ASPC/NMA/PCNA Guideline for the Prevention, Detection, Evaluation, and Management of High Blood Pressure in Adults: A Report of the American College of Cardiology/American Heart Association Task Force on Clinical Practice Guidelines. J Am Coll Cardiol. 2017 Nov 7. pii: S0735-1097(17)41519-1. doi: 10.1016/j.jacc.2017.11.006
40. Huggett DL, Elliott ID, Overend TJ, Vandervoort AA. Comparison of heart-rate and blood-pressure increases during isokinetic eccentric versus isometric exercise in older adults. J Aging Phys Act. 2004;12(2):157-169.
41. Vallejo AF, Schroeder ET, Zheng L, Jensky NE, Sattler FR. Cardiopulmonary responses to eccentric and concentric resistance exercise in older adults. Age Ageing. 2006;35(3):291-297.
42. Rezk CC, Marrache RC, Tinucci T, Mion D Jr, Forjaz CL. Post-resistance exercise hypotension, hemodynamics, and heart rate variability: influence of exercise intensity. Eur J Appl Physiol. 2006;98(1):105-112.
43. Borg E, Borg G. A comparison of AME and CR100 for scaling perceived exertion. Acta Psychol (Amst). 2002;109(2):157-175.
44. The Food Standards Agency (2002). McCance and Widdowson’s the Composition of Foods Integral Dataset (6th Summary Edition). Available on line: http://webarchive.nationalarchives.gov.uk. Accessed in: August 20, 2017

# Useful readings

1. Research Governance Framework for Health and Social Care, 2nd edition 2005
2. Health Research Authority: Progress and Safety Reporting - http://www.hra.nhs.uk/resources/during-and-after-your-study/progress-and-safety-reporting/
3. MHRA: Notify about a device investigation - https://www.gov.uk/notify-mhra-about-a-clinical-investigation-for-a-medical-device
4. MHRA: Medical devices: guidance for manufacturers on vigilance - <https://www.gov.uk/government/collections/medical-devices-guidance-for-manufacturers-on-vigilance>

# Appendices

# Appendix 1

THE SARC-F QUESTIONNAIRE

**S**trength: How much difficulty do you have in lifting and carrying 10 pounds / a bag of shopping?

None: 0

Some: 1

A lot or unable: 2

**A**ssistance in walking: How much difficulty do you have walking across a room?

None: 0

Some: 1

A lot, use aids, or unable: 2

**R**ise from a chair: How much difficulty do you have transferring from a chair or bed?

None: 0

Some: 1

A lot or unable without help: 2

**C**limb stairs: How much difficulty do you have climbing a flight of 10 stairs?

None: 0

Some: 1

A lot or unable: 2

**F**alls: How many times have you fallen in the past year?

None: 0

1 to 3 falls: 1

4 or more falls: 2

______________________________________________________________________________________________________________

**Appendix 2**

R100® scale^43^

**Instructions for participants** (explained to participants by exercise physiologist)

*Assessment ~10 minutes after exercise*

- - Using the scale, we would like you to rate your perceptions of EFFORT, that is, how difficult the session felt to you.
  - Your perception of EFFORT should be a conscious awareness of how hard (or easy) the whole session was. It shouldn’t be influenced your feelings of fatigue, pain or discomfort (try separate these from effort as best you can).
  - You will be asked to rate your perceptions of overall effort, and then separate scores for upper-body muscle effort and lower-body muscle effort.
  - For upper-body muscle RPE, the perception of exertion depends mainly on the strain and exertion in your arm muscles during training.
  - For lower-body muscle RPE, the perception of exertion depends mainly on the strain and exertion in your leg muscles during training.
  - Your scores should reflect the whole session, which is all exercises that you completed after the warm-up.
  - Try to appraise your feelings as honestly, without thinking about what the actual training was or should have been.

**Appendix 3**

Visual analogue scale for muscle soreness


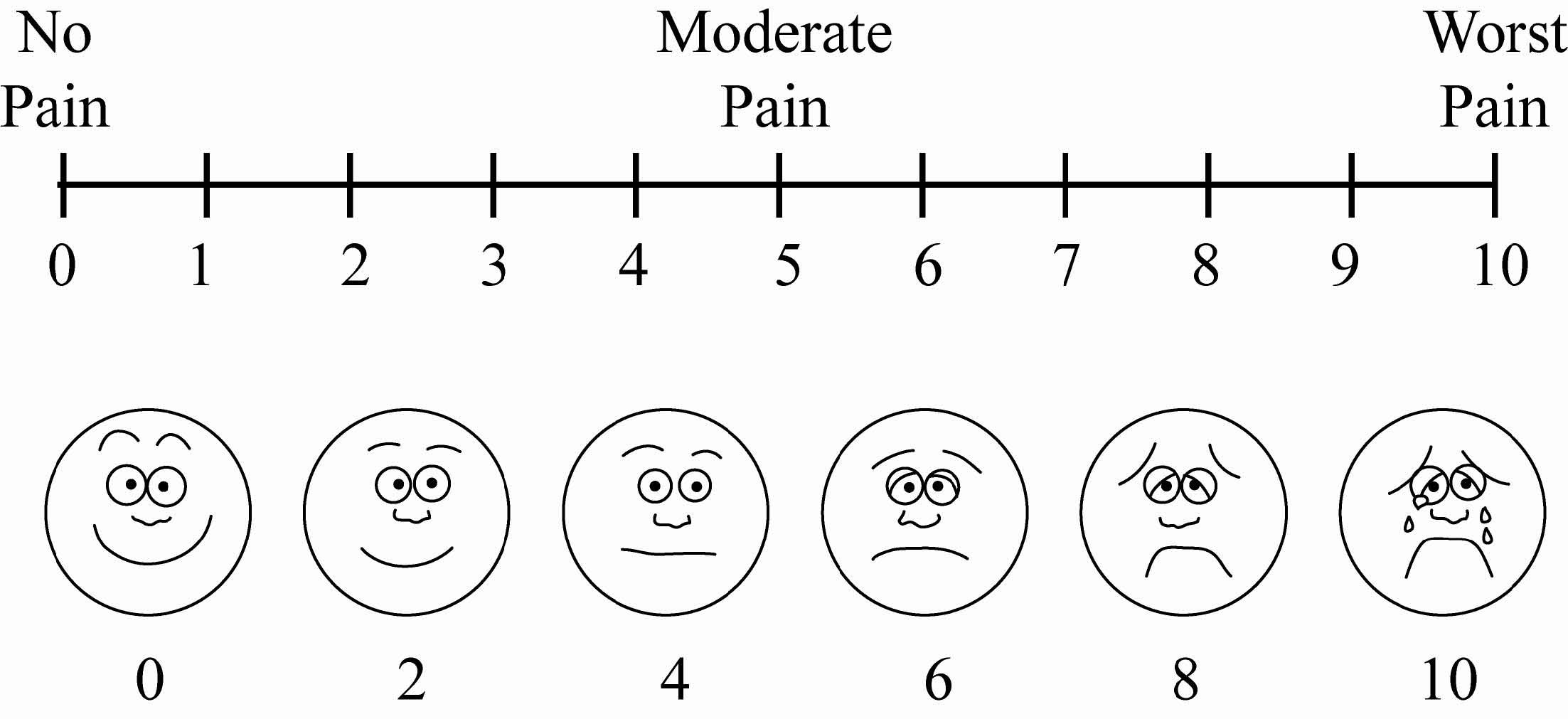


0 = no pain, 1-3 = mild pain, 4-6 = moderate pain; 7-10 = severe pain

**Instructions for participants** (explained to participants by a researcher)

- This is a Visual Analogue Scale. The scale describes the intensity of your pain, 0 meaning no pain at all, 1 to 3 meaning mild pain, 4-6 moderate pain, and 7-10 the worst pain.
- Assessment ~45 minutes after exercise: Which number on the scale describes the best your muscle soreness in your (i) arms and (ii) legs?
- *Assessment in the evening over the phone (6-7 hours after exercise)*: A researcher will call you in the evening after each visit to the Parks to ask you again about your muscle soreness in your arms and legs. Please use the visual analogue scale that we gave you to rate your muscle soreness in your (i) arms and (ii) legs.
